# The malate shuttle detoxifies ammonia in exhausted T cells by producing 2-ketoglutarate

**DOI:** 10.1038/s41590-023-01636-5

**Published:** 2023-10-09

**Authors:** Nina Weisshaar, Sicong Ma, Yanan Ming, Alaa Madi, Alessa Mieg, Marvin Hering, Ferdinand Zettl, Kerstin Mohr, Nora Ten Bosch, Diana Stichling, Michael Buettner, Gernot Poschet, Glynis Klinke, Michael Schulz, Nina Kunze-Rohrbach, Carolin Kerber, Isabel Madeleine Klein, Jingxia Wu, Xi Wang, Guoliang Cui

**Affiliations:** 1 T Cell Metabolism Group (D192), German Cancer Research Center (DKFZ), Heidelberg, Germany; 2 Institute of Health and Medicine, Hefei Comprehensive National Science Center, Hefei, China; 3 Faculty of Biosciences, Heidelberg University, Heidelberg, Germany; 4 Helmholtz Institute for Translational Oncology (HI-TRON)—A Helmholtz Institute of the DKFZ, Mainz, Germany; 5 Metabolomics Core Technology Platform, Centre for Organismal Studies (COS), Heidelberg University, Heidelberg, Germany; 6 Tissue Bank of the German Center for Infection Research (DZIF), Partner Site Heidelberg, Institute of Pathology, Heidelberg University Hospital, Heidelberg, Germany; 7 State Key Laboratory of Reproductive Medicine and Offspring Health, Nanjing Medical University, Nanjing, China

**Keywords:** Cellular immunity, Enzymes, CD8-positive T cells

## Abstract

The malate shuttle is traditionally understood to maintain NAD^+^/NADH balance between the cytosol and mitochondria. Whether the malate shuttle has additional functions is unclear. Here we show that chronic viral infections induce CD8^+^ T cell expression of GOT1, a central enzyme in the malate shuttle. *Got1* deficiency decreased the NAD^+^/NADH ratio and limited antiviral CD8^+^ T cell responses to chronic infection; however, increasing the NAD^+^/NADH ratio did not restore T cell responses. *Got1* deficiency reduced the production of the ammonia scavenger 2-ketoglutarate (2-KG) from glutaminolysis and led to a toxic accumulation of ammonia in CD8^+^ T cells. Supplementation with 2-KG assimilated and detoxified ammonia in *Got1*-deficient T cells and restored antiviral responses. These data indicate that the major function of the malate shuttle in CD8^+^ T cells is not to maintain the NAD^+^/NADH balance but rather to detoxify ammonia and enable sustainable ammonia-neutral glutamine catabolism in CD8^+^ T cells during chronic infection.

## Abstract (web summary)

The malate shuttle is understood to control the NAD^+^/NADH balance. Here the authors show that GOT1, a central enzyme in the malate shuttle, rather promotes antiviral CD8^+^ T cell responses by detoxifying ammonia.

## Main

CD8^+^ T cells have crucial functions in the defense against infectious diseases. After infection, antigen-specific naïve CD8^+^ T cells undergo clonal expansion and differentiate into anti-infection effector T (T_eff_) cells. In acute infection, CD8^+^ memory T (T_mem_) cells gradually mature after antigen clearance and provide long-term protection against reinfection. In chronic infection, CD8^+^ T cells become functionally exhausted and are referred to as exhausted T (T_ex_) cells^1^. These T cell subsets have distinct metabolic characteristics in glycolysis and oxidative phosphorylation^2,3^. The malate shuttle indirectly transports glycolysis-produced nicotinamide adenine dinucleotide hydrogen (NADH) into the mitochondria, where NADH is converted to its oxidized form, nicotinamide adenine dinucleotide (NAD^+^), by oxidative phosphorylation^4^. Whether and how the malate shuttle regulates CD8^+^ T_ex_ cell differentiation is unknown.

A key enzyme in the malate shuttle is glutamic-oxaloacetic transaminase 1 (GOT1, or aspartate aminotransferase), which generates oxaloacetate and glutamate from 2-ketoglutarate (2-KG) and aspartate. Pharmacological inhibition of GOT1 with aminooxyacetate (AOA) affects T cell proliferation^5^. However, evidence has indicated that AOA is a pan inhibitor of pyridoxal phosphate-dependent enzymes^6–8^, thus suggesting that AOA might not be suitable to specifically and accurately assess the biological function of GOT1. We used a mouse strain with T cell-specific ablation of *Got1*. We found that GOT1 catalyzed an atypical transamination chemical reaction to produce the ammonia scavenger 2-KG. CD8^+^ T cells required GOT1 to detoxify ammonia and to catabolize glutamine in an ammonia-neutral manner when mitochondrial respiration is inhibited by chronic infection.

## T cell receptor (TCR) stimulation induces GOT1 expression in CD8^+^ T cells

**Fig. 1:**
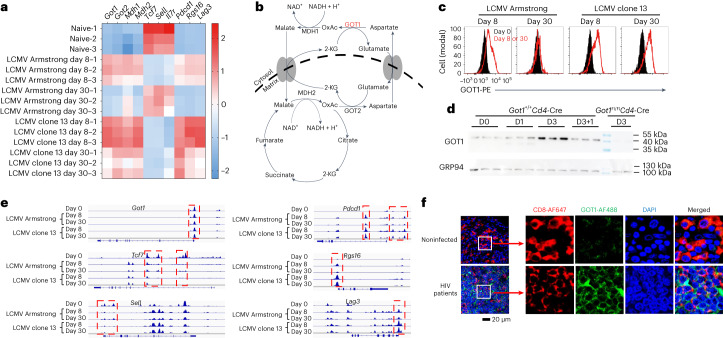
Antigenic stimulation induces GOT1 expression in human and mouse CD8^+^ T cells. **a**, Heat maps show the mRNA expression *z* scores of the indicated genes in splenic LCMV GP_33–41_ epitope-specific P14 CD8^+^ T cells purified from C57BL/6 host mice after LCMV Armstrong or LCMV clone 13 infection. **b**, Illustration of the malate shuttle and TCA cycle-associated biochemical reactions. **c**, Flow cytometry histograms in red show GOT1 protein expression in P14 CD8^+^ T cells purified from C57BL/6 host mice killed at the indicated time points after LCMV infection. Filled histograms in black ‘day 0’ represent P14 CD8^+^ T cells from naïve mice and show the basal levels of GOT1 protein expression. *n* = 6 mice at each time point. **d**, *Got1*^+/+^*Cd4-*Cre P14 splenocytes (*Got1*-sufficient) were cultured with GP_33–41_ peptide for 1 d (D1) or 3 d (D3) before western blot analysis. On day 3, the cells were washed and cultured with IL-15 (10 ng ml^−^^1^) for another day (D3 + 1). *Got1*^Fl/Fl^*Cd4-*Cre P14 splenocytes (*Got1*-deficient) were included as negative controls. GRP94 was used as a loading control. *n* = 3 mice per group. Uncropped immunoblot images are shown in Source Data Fig. 8. **e**, ATAC-sequencing signal profiles across *Got1*, *Tcf7*, *Sell*, *Pdcd1*, *Rgs16* and *Lag3* loci in P14 CD8^+^ T cells collected from naïve mice or mice infected with LCMV Armstrong or LCMV clone 13. Differentially accessible peaks are highlighted in red. Two mice were pooled in a single sample to obtain sufficient cells for analysis in the group of ‘LCMV clone 13 infection day 30’. Six mice were used in total in this group. Three mice were used in each of the other groups (**a**,**e**). **f**, Immunofluorescence microscopy images show the expression of human GOT1 in CD8^+^ T cells in lymph node sections from HIV-infected patients or donors without HIV infection. Data are representative of ten images of six donors from three independent experiments. Six-week-old male mice were used (**a**,**c**,**d**,**e**). Source data

To examine the potential role of the malate shuttle in antiviral CD8^+^ T cell responses, we monitored the expression of malate shuttle-associated genes in CD8^+^ T cells collected from mice infected with lymphocytic choriomeningitis virus (LCMV) Armstrong, which induces transient infection and acute CD8^+^ T cell responses, or LCMV clone 13 strain, which induces persistent infection and chronic CD8^+^ T cell responses^9^. Eight days after infection, both LCMV strains showed increased expression of genes associated with CD8^+^ T cell activation, such as *Pdcd1*, *Rgs16* and *Lag3*, in agreement with previous findings^9^. In contrast, genes associated with T cell stemness and quiescence, such as *Tcf7*, *Sell* and *Il7r*, were expressed at lower levels in CD8^+^ T_eff_ cells than in naïve CD8^+^ T cells (Fig. 1a). The mRNA expression levels of genes involved in the malate shuttle, such as *Got1*, *Got2*, *Mdh1* and *Mdh2*, increased as naïve CD8^+^ T cells differentiated into CD8^+^ T_eff_ cells (Fig. 1a,b). Furthermore, when CD8^+^ T_eff_ cells matured into CD8^+^ T_mem_ cells 30 d after LCMV Armstrong infection, the expression of the malate shuttle-associated genes decreased to levels comparable to those in naïve T cells, and this response was accompanied by decreased expression of *Pdcd1*, *Rgs16* and *Lag3* and reexpression of *Tcf7*, *Sell* and *Il7r*. Persistent LCMV clone 13 infections induced and maintained the expression of *Got1*, *Got2*, *Mdh1* and *Mdh2*, in an expression pattern resembling that of *Pdcd1*, *Rgs16* and *Lag3* (Fig. 1a). The kinetics of GOT1 protein levels in virus-specific CD8^+^ T cells was similar to that of *Got1* mRNA (Fig. 1c). Similar to the in vivo observations, TCR-transgenic P14 CD8^+^ T cells showed significantly elevated GOT1 protein expression 3 d after cognate peptide GP_33–41_ stimulation in vitro (Fig. 1d). GOT1 protein levels decreased when the GP_33–41_ peptide was washed out and replaced with interleukin (IL)-15, thus suggesting that antigen persistence was required for maintaining GOT1 expression.

**Extended Data Fig. 1:**
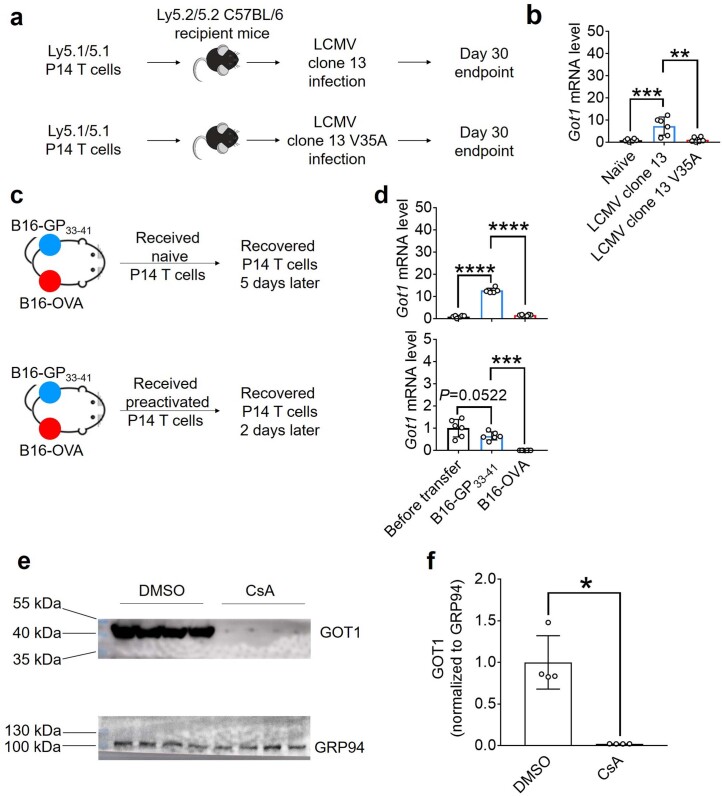
Antigenic stimulation induces GOT1 expression in CD8^+^ T cells. **a**, Illustration of the experimental design of **b**. **b**, A bar graph shows *Got1* mRNA expression in P14 T cells recovered from host mice infected with LCMV clone 13 or LCMV clone 13 V35A (the valine residue at position 35 was replaced with alanine), as quantified by qRT-PCR analysis. **c**, Illustration of the experimental design of **d**. **d**, A bar graph shows *Got1* mRNA expression in P14 T cells before transfer and in P14 T cells recovered from tumors, as quantified by qRT-PCR analysis. **e**,**f**, P14 splenocytes were cultured with the GP_33–41_ peptide for 3 days with or without cyclosporin A (CsA, 5 µM) before western blot analysis (**e**). The bar graph shows the results of densitometric quantification of the immunoblot bands (**f**). GRP94 was used as a loading control. The results are presented as mean ± s.d. *P<0.05; **P<0.01; ***P<0.001; ****P<0.0001. Comparisons were performed with one-way ANOVA (**b** and **d**) or the two-tailed Mann-Whitney test (**f**, data points were not normally distributed). N = 6 mice (**b** and **d**) or N = 4 (**e**–**f**) in each group. *P* values in **b** (from left to right): 0.0009, 0.001; in **d** (top): both P values <1.0 × 10^−15^; in **d** (bottom): 0.0522, 0.0009; in **f**: 0.0286. Seven-week-old female mice were used (**b**, **d**, **e**, **f**). Source data

To further examine the potential role of TCR stimulation in driving *Got1* expression, we infected C57BL/6 mice with LCMV clone 13 or a mutated strain LCMV clone 13 V35A^10^. P14 CD8^+^ T cells recognize the GP_33–41_ epitope of LCMV clone 13, but not the mutated GP_33–41_ V35A epitope of LCMV clone 13 V35A. P14 CD8^+^ T cells in mice infected with LCMV clone 13 expressed higher levels of *Got1* than those in mice infected with LCMV clone 13 V35A, suggesting that TCR stimulation drives *Got1* expression (Extended Data Fig. 1a,b). Furthermore, we implanted C57BL/6 mice with B16 melanoma cells expressing the GP_33–41_ epitope (B16-GP_33–41_) or the OVA epitope (B16-OVA)^11^. Subsequently, we adoptively transferred GP_33–41_ epitope-specific P14 T cells into the tumor-bearing mice. P14 CD8^+^ tumor-infiltrating lymphocytes recovered from B16-GP_33–41_ tumors expressed significantly higher levels of *Got1* than those recovered from B16-OVA tumors, indicating that TCR stimulation induced *Got1* expression (Extended Data Fig. 1c,d).

**Extended Data Fig. 2:**
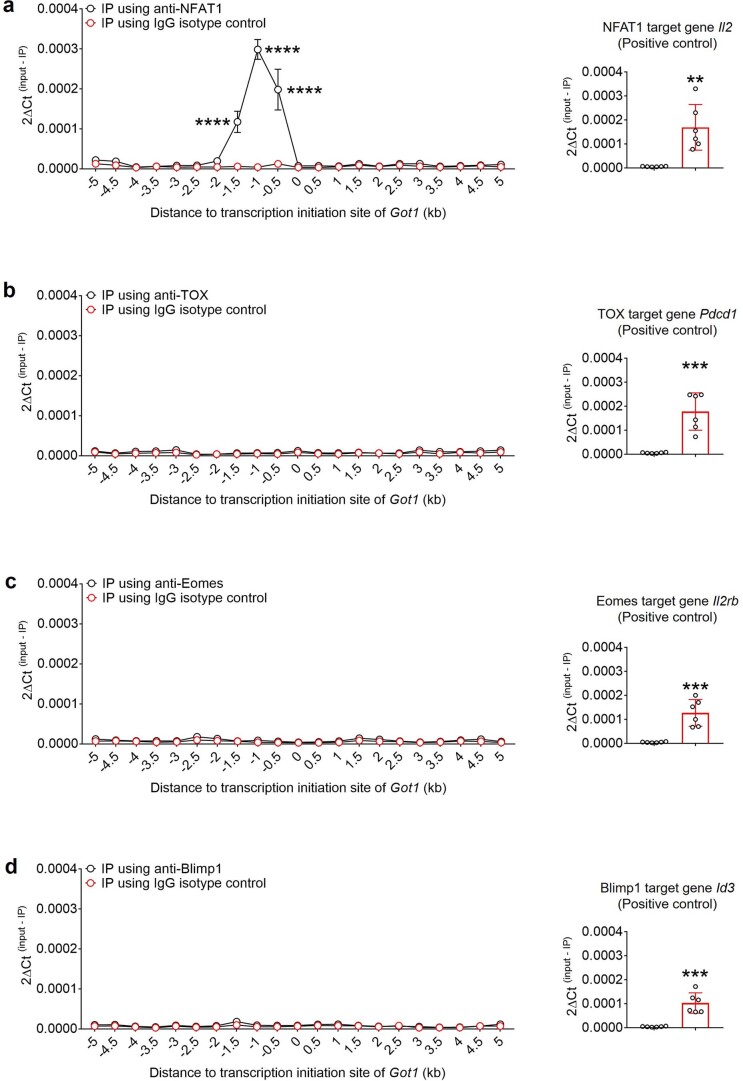
NFAT1 binds to *Got1* locus. **a**–**d**, P14 splenocytes were cultured with GP_33–41_ peptide for 3 days before ChIP-PCR analysis. PCR was performed using primers evenly spaced across a 10kb region of *Got1* locus. Line curves show the relative quantification results of DNA fragments pulled down using indicated antibodies or IgG isotype control. Bar graphs show the binding of NFAT1 to *Il2* (a known target gene of NFAT1) locus, the binding of TOX to *Pdcd1* (a known target gene of TOX) locus, the binding of Eomes to *Il2rb* (a known target gene of Eomes) locus, and the binding of Blimp1 to *Id3* (a known target gene of blimp1) locus. The data are presented as mean ± s.d. **P<0.01; ***P<0.001; ****P<0.0001. Comparisons were performed with two-way ANOVA (line graphs, **a**–**d**), a two-tailed Student’s *t*-test (bar graphs, **a**–**d**, data points were normally distributed). *P* values in **a** (from left to right): 9.1 × 10^−13^, <1.0 × 10^−15^, <1.0 × 10^−15^, 0.002; **b**: 0.0003; **c**: 0.0003; in **d**: 0.0002. Seven-week-old male mice were used (**a**–**d**). Source data

Inhibition of the NFAT pathway significantly reduced GOT1 protein expression (Extended Data Fig. 1e,f). To test whether *Got1* is a target gene of NFAT, or other transcription factors known to regulate T cell differentiation, such as TOX, Eomes, and Blimp1, we performed chromatin immunoprecipitation (ChIP)-PCR analysis. NFAT1, TOX, Eomes and Blimp1 are known to bind to the genetic loci of *Il2* (ref. ^12^), *Pdcd1* (ref. ^13^), *Il2rb*^14^ and *Id3* (ref. ^15^), respectively. NFAT1, but not the other three transcription factors directly bound to the −1.5 to −0.5 kb region of the *Got1* locus (Extended Data Fig. 2a–d). Taken together, these results suggested that NFAT1 bound to the *Got1* locus and promoted its expression.

The transcription initiation site (TIS) of *Got1* was constantly accessible before and after infections (Fig. 1e), thereby suggesting that LCMV infection induced the expression of *Got1* at the transcriptional level but not the epigenetic level. This mode of regulation of *Got1* expression differed from that of *Tcf7*, *Sell*, *Pdcd1*, *Rgs16* and *Lag3* (Fig. 1e). The longitudinal dynamics of chromatin accessibility of the TISs of *Sell*, *Rgs16* and *Lag3* and intron regions or intergenic regions in the *Tcf7* and *Pdcd1* loci, previously shown to be influenced by infection^16,17^, mimicked their gene expression levels during the course of LCMV infection.

GOT1 protein was readily detectable in CD8^+^ T cells from HIV-infected patients. Through confocal microscopic analysis, we observed that CD8^+^ T cells in lymph node sections from HIV-infected patients, but not those from noninfected donors, expressed GOT1 protein (Fig. 1f). Collectively, these results suggested that both human and mouse CD8^+^ T cells expressed GOT1 during chronic infections.

## Antiviral CD8^+^ T cell responses require GOT1 protein

**Extended Data Fig. 3:**
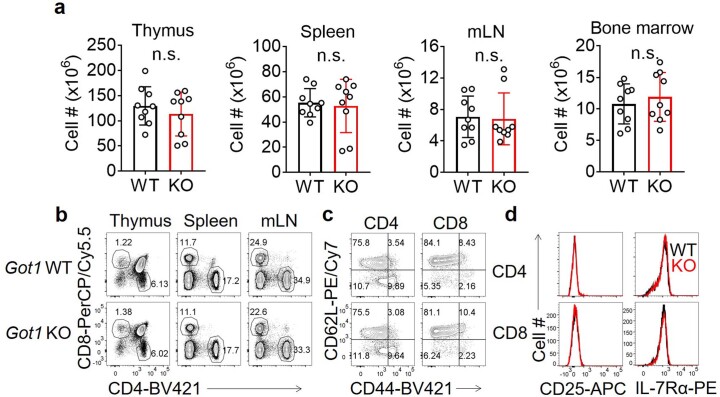
Basic immune characterization of *Got1*-deficient mice. **a**, Bar graphs show the numbers of thymocytes, splenocytes, mesenteric lymphocytes (mLNs), and bone marrow cells in littermates. **b**, Flow cytometry dot plots show the percentages of CD4^+^ and CD8^+^ T cells in thymocytes, mLNs, and splenocytes. **c**, The percentages of splenic CD4^+^ and CD8^+^ T cells expressing CD44 and CD62L are shown. **d**, Flow cytometry histograms show the expression of CD25 and IL-7Rα in splenic CD4^+^ and CD8^+^ T cells. The data are expressed as mean ± s.d. and are cumulative (**a**) or representative (**b**–**d**) data from two independent experiments with nine pairs of littermates. n.s., not significant. Comparisons were performed with a two-tailed Student’s *t*-test (**a**, data points were normally distributed). *P* values in **a** (from left to right): 0.414, 0.751, 0.852, 0.516. Six to seven-week-old female mice were used (**a**–**d**). Source data

To examine the potential role of GOT1 in antiviral CD8^+^ T cell responses, we created a mouse model with T cell-specific ablation of *Got1* by breeding *Got1*^Flox/Flox^ mice with the *Cd4*-Cre strain. Because CD4^+^CD8^+^ thymocytes give rise to mature peripheral CD4^+^ T cells and CD8^+^ T cells, *Cd4*-driven Cre deletes LoxP-flanked genes in both CD4^+^ T cells and CD8^+^ T cells^18,19^. We confirmed that the GOT1 protein was deleted through western blot analysis (Fig. 1d). *Got1*-deficient (knockout (KO)) mice had similar numbers of thymocytes, splenocytes, lymphocytes and bone marrow cells to those observed in their wild-type (WT) littermates (Extended Data Fig. 3a). *Got1* deficiency did not significantly alter the CD4^+^ and CD8^+^ T cell percentages among the thymocyte, splenocyte and lymphocyte populations (Extended Data Fig. 3b). Moreover, *Got1* KO and WT mice had comparable protein levels of CD44, CD62L, CD25 and IL-7Rα (Extended Data Fig. 3c–d). Collectively, *Got1* deficiency did not influence either thymic T cell development or peripheral T cell homeostasis under steady-state conditions.

**Fig. 2:**
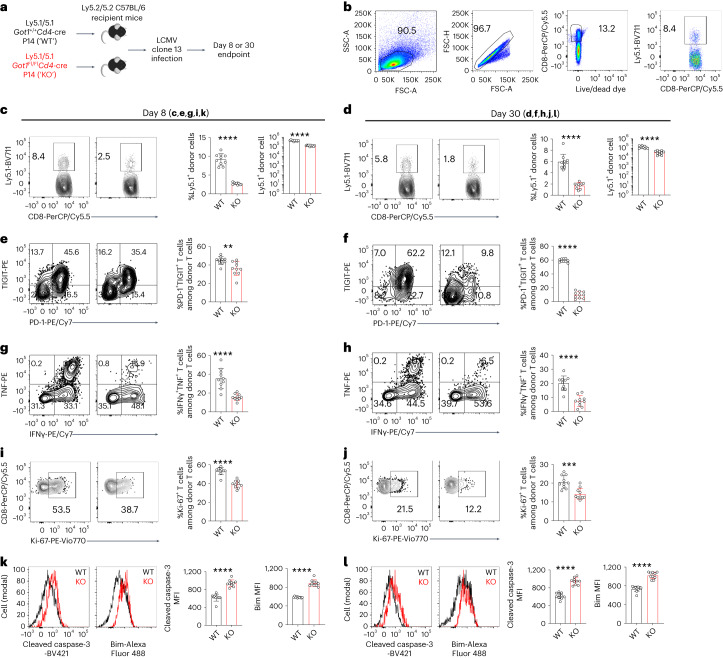
Antiviral CD8^+^ T cell responses require GOT1 during chronic infection. **a**, Illustration of the experimental design. **b**, Fluorescence-activated cell sorting (FACS) gating strategies used in **c**–**l**. **c**–**l**, Contour plots, histograms and bar graphs show the flow cytometry staining results of Ly5.1^+^ donor P14 CD8^+^ T cells (**c**,**d**), PD-1 and TIGIT (**e**,**f**), cytokines (**g**,**h**), Ki-67 (**i**,**j**) and cleaved caspase-3 and Bim (**k**,**l**) in *Got1*-deficient and sufficient P14 CD8^+^ T cells. Cells were stimulated with GP_33–41_ peptides before the flow cytometry staining (**g**–**h**). Data were pooled from two independent experiments (**c**–**l**) with ten C57BL/6 mice in each group receiving *Got1*-deficient and sufficient donor P14 CD8^+^ T cells. The results are presented as mean ± s.d. **P < 0.01; ***P < 0.001; ****P < 0.0001. Comparisons were performed with the two-tailed Mann–Whitney test (percentage of Ly5.1^+^ donor cells in **d** and **i**, cleaved caspase-3 MFI in **k** and Bim MFI in **l**; data points were not normally distributed) or a two-tailed Student’s *t*-test (other comparisons). In **c**, *P* = 1.14 × 10^−10^ (left) and *P* = 9.48 × 10^−12^ (right); in **d**, *P* = 1.08 × 10^−5^ (left) and *P* = 9.08 × 10^−7^ (right); in **e**, *P* = 0.0048; in **f**, *P* = 1.0 × 10^−15^; in **g**, *P* = 2.47 × 10^−5^; in **h**, *P* = 6.99 × 10^−6^; in **i**, *P* = 2.17 × 10^−5^; in **j**, *P* = 0.0003; in **k**, *P* = 1.08 × 10^−5^ (left) and *P* = 8.84 × 10^−11^ (right); in **l**, *P* = 8.70 × 10^−9^ (left) and *P* = 1.08 × 10^−5^ (right). Six-week-old female mice were used (**b**–**l**). Source data

To examine the potential role of GOT1 in antiviral CD8^+^ T cell responses, we adoptively transferred *Got1* KO or WT P14 CD8^+^ T cells into C57BL/6 host mice before LCMV clone 13 infection (Fig. 2a). The percentages of *Got1* KO donor T cells among the total CD8^+^ T cells in the C57BL/6 host mice, as well as the absolute numbers of *Got1* KO donor T cells, were lower than those of *Got1* WT donor T cells at day 8 and day 30 after infection (Fig. 2b–d). *Got1* KO T cells expressed lower levels of inhibitory receptors, such as PD-1 and TIGIT than WT cells (Fig. 2e,f), suggesting that PD-1^high^ and TIGIT^high^ CD8^+^ T cells were more dependent than PD-1^low^ and TIGIT^low^ CD8^+^ T cells on GOT1. *Got1* deficiency also decreased the production of effector cytokines, IFNγ and TNF (Fig. 2g,h). Ki-67 protein levels were lower in *Got1* KO than WT CD8^+^ T cells (Fig. 2i,j), thus suggesting that virus-specific CD8^+^ T cells required GOT1 to proliferate during LCMV clone 13 infection. *Got1* deficiency increased the protein levels of cleaved caspase-3 and Bim (Fig. 2k,l), two proteins positively correlated with apoptosis, thereby suggesting that GOT1 promoted the survival of CD8^+^ T cells. Together, these results indicated that GOT1 was indispensable for functional CD8^+^ T cell survival and proliferation in the presence of persistent antigenic stimulation.

## 2-KG decreases the concentration of ammonia In CD8^+^ T cells

**Fig. 3:**
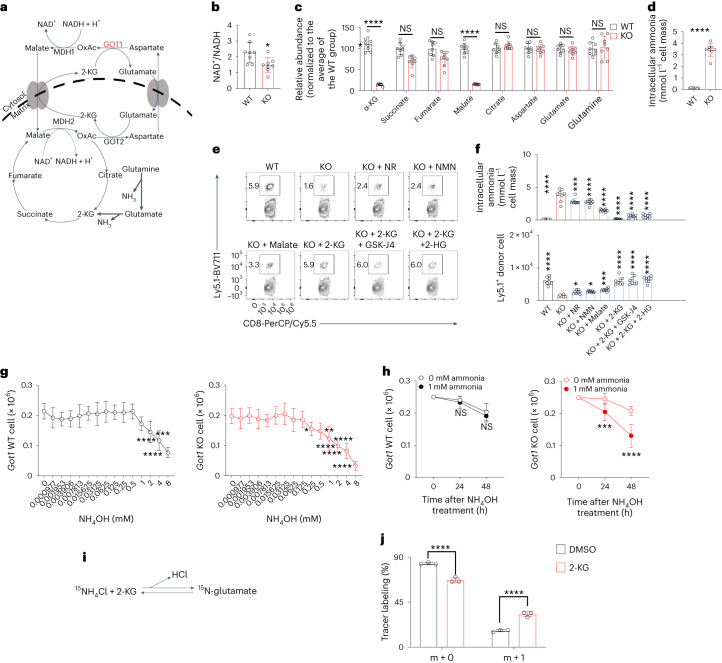
T_ex_ cells require GOT1 to accumulate 2-KG that detoxifies ammonia. **a**, The malate shuttle, TCA cycle and glutaminolysis. **b**–**d**, Bar graphs show NAD^+^/NADH ratios (**b**), indicated metabolites (**c**) and ammonia (**d**) in *Got1*-deficient and sufficient donor P14 CD8^+^ T cells isolated from C57BL/6 host mice infected 30 d earlier with LCMV clone 13. **e**,**f**, Splenocytes were isolated from host mice 30 d after infection and were cultured with GP_33–41_ peptides in the presence of the indicated compounds for 1 d. Flow cytometry contour plots show the percentages of Ly5.1^+^ donor T cells among CD8^+^ T cells (**e**). Bar graphs show the concentrations of ammonia in donor T cells and the numbers of T cells (**f**). **g**,**h**, *Got1*-deficient and *Got1*-sufficient donor P14 CD8^+^ T cells were isolated from C57BL/6 host mice infected with LCMV clone 13 30 d earlier. 0.25 × 10^6^ cells were cultured with anti-CD3 and anti-CD28 in the presence or absence of NH_4_OH for 2 d. Line graphs show the number of cells after culturing with NH_4_OH at the indicated concentrations (**g**) or for the indicated time (**h**). **i**, Illustration of the incorporation of the tracer ^15^N from ^15^NH_4_Cl into ^15^N-glutamate in *Got1*-sufficient donor P14 CD8^+^ T cells. ‘m + 0’ or ‘m + 1’ indicates glutamate with zero or one ^15^N atom. **j**, A bar graph shows amounts of ^15^N-glutamate. Data are combined from two experiments with eight (**b**–**d**,**f**–**h**) or three (**j**) mice. The data are presented as mean ± s.d. *P < 0.05; **P < 0.01; ***P < 0.001; ****P < 0.0001. Comparisons were performed with a two-tailed Student’s *t* test (**b**,**d**; data points were normally distributed), two-way ANOVA (**c**,**h**,**j**) and one-way ANOVA (**f**,**g**). In **b**, *P* = 0.0131; in **c** (from left to right), *P* = 1.4 × 10^−14^, *P* = 0.071, *P* = 0.713, *P* = 6.8 × 10^−14^, *P* = 0.207, *P* = 0.860, *P* = 0.991 and *P* = 0.671; in **d**, *P* = 1.0 × 10^−10^; in **f** (top), *P* = 1.0 × 10^−15^, *P* = 0.000403208, *P* = 1.0 × 10^−15^, *P* = 1.0 × 10^−15^, *P* = 1.0 × 10^−15^, *P* = 1.0 × 10^−15^, *P* = 1.0 × 10^−15^ and (bottom) *P* = 1.0 × 10^−15^, *P* = 0.048, *P* = 0.021, *P* = 0.0005, *P* = 1.0 × 10^−15^, *P* = 1.0 × 10^−15^, *P* = 1.0 × 10^−15^; in **g**, *P* = 0.0001, *P* = 1.0 × 10^−15^, *P* = 1.0 × 10^−15^, *P* = 0.040, *P* = 0.009, *P* = 1.0 × 10^−15^, *P* = 1.0 × 10^−15^, *P* = 1.0 × 10^−15^ and *P* = 1.0 × 10^−15^; in **h**, *P* = 0.7694, *P* = 0.373, *P* = 0.0009 and *P* = 5.438 × 10^−9^; in **j**, both *P* = 1.2 × 10^−5^. Six-week-old female mice were used (**b**–**h**,**j**). NS, not significant. Source data

We measured NAD^+^, NADH and other metabolites associated with the malate shuttle (Fig. 3a–d). The NAD^+^/NADH ratio was decreased by *Got1* deficiency in virus-specific CD8^+^ T cells (Fig. 3b), in agreement with the widely appreciated role of the malate shuttle in maintaining the NAD^+^/NADH balance. Supplementation with the NAD^+^ precursor molecules nicotinamide riboside (NR) and nicotinamide mononucleotide (NMN) did not fully restore the cell numbers of *Got1* KO CD8^+^ T cells (Fig. 3e,f), suggesting that although *Got1* deficiency disturbed the NAD^+^/NADH balance, it was not primarily responsible for defects in *Got1* KO CD8^+^ T cell accumulation.

Furthermore, *Got1* deficiency decreased the abundance of 2-KG and malate in CD8^+^ T cells (Fig. 3c). Because these metabolites have been shown to regulate ammonia metabolism^20,21^, we measured ammonia and observed that *Got1* deficiency significantly increased concentrations of ammonia (Fig. 3d). We quantified the mass of CD8^+^ T cells by using graduated packed cell volume tubes. On the basis of the assumption that the major component of cells was water, the average concentrations of ammonia in *Got1*-deficient CD8^+^ T cells exceeded 3,000 µM (calculated by dividing the total amount of ammonia by the CD8^+^ T cell mass). The physiological concentrations of ammonium in mouse blood range from 23.8 to 76.9 µM^22^. Because ammonia has been reported to be toxic at concentrations above 1,000 µM^23^, we hypothesized that the failure of ammonia removal caused *Got1* KO CD8^+^ T cell death. Supplementation with cell membrane-permeable 2-KG and malate, particularly 2-KG, decreased the abundance of ammonia and increased the percentages of *Got1* KO CD8^+^ T cells among all CD8^+^ T cells (Fig. 3f). Moreover, 2-KG has been shown to activate histone demethylase^24,25^ and DNA demethylase^26,27^. GSK-J4 and 2-HG, which are inhibitors of histone demethylase and DNA demethylases^28–30^, did not influence the pro-survival and ammonia-decreasing effects of 2-KG (Fig. 3e,f). These results suggested that 2-KG’s restoration of *Got1* KO CD8^+^ T cell accumulation did not occur through activating histone and DNA demethylases.

**Extended Data Fig. 4:**
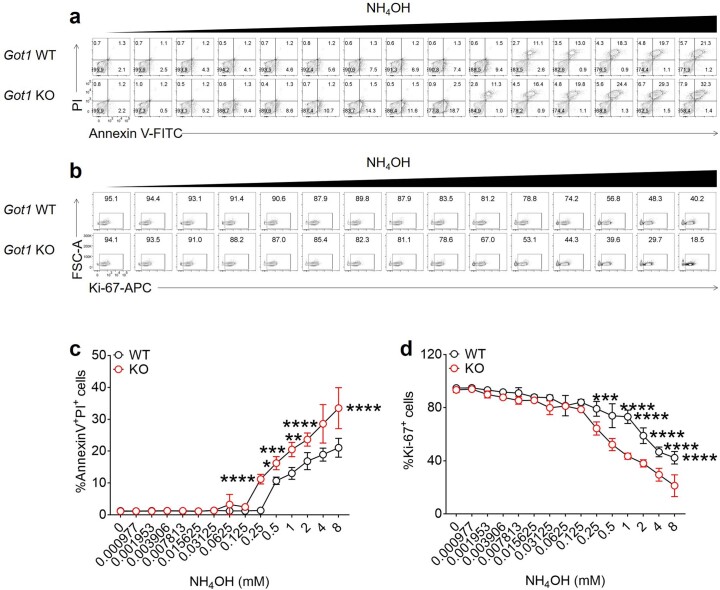
Ammonia promotes T cell apoptosis and inhibits T cell proliferation. **a**–**d**, *Got1*-deficient and -sufficient donor P14 CD8^+^ T cells were isolated from C57BL/6 host mice infected with LCMV Armstrong 8 days earlier. A total of 0.25 × 10^6^ cells were cultured with anti-CD3 and anti-CD28 in the presence or absence of NH_4_OH for 2 days. FACS plots (**a**–**b**) and line graphs (**c**–**d**) show the percentages of AnnexinV^+^ PI^+^ cells (**a**, **c**) and Ki-67^+^ cells (**b**, **d**). Data are combined from two experiments with three mice in total. The results are presented as mean ± s.d. *P<0.05; **P<0.01; ***P<0.001; ****P<0.0001. Comparisons were performed using two-way ANOVA. *P* values in **c** (from left to right): 3.6 × 10^−6^, 0.026, 0.0006, 0.003, 5.3 × 10^−6^, 9.7 × 10^−9^; in **d**: 0.0006, 2.3 × 10^−7^, 1.9 × 10^−11^, 6.3 × 10^−7^, 4.0 × 10^−5^, 4.8 × 10^−7^. Six-week-old female mice were used (**a**–**d**). Source data

Ammonia significantly inhibited the growth of *Got1* WT CD8^+^ T cells and *Got1* KO CD8^+^ T cells at concentrations higher than 2 mM or 0.25 mM, respectively (Fig. 3g). Ammonia (at 1 mM) did not significantly influence *Got1* WT CD8^+^ T cell numbers at either 24 h or 48 h after treatment (Fig. 3h). In contrast, ammonia at the same concentration significantly decreased *Got1* KO CD8^+^ T cell numbers. Ammonia treatment increased the percentages of Annexin V^+^ propidium iodide (PI)^+^ CD8^+^ T cells (Extended Data Fig. 4a,c), suggesting that ammonia promotes CD8^+^ T cell apoptosis. Additionally, ammonia inhibited the expression levels of Ki-67, and *Got1*-deficient CD8^+^ T cells were more susceptible to ammonia-induced inhibition of cell proliferation than *Got1*-sufficient CD8^+^ T cells (Extended Data Fig. 4b,d). Taken together, these results suggested that ammonia promoted CD8^+^ T cell apoptosis and inhibited cell proliferation, and *Got1* deficiency further sensitized CD8^+^ T cells to ammonia-induced apoptosis and inhibition of cell proliferation.

To examine whether 2-KG assimilated free ammonia into glutamate and, therefore, detoxified free ammonia (Fig. 3i), we cultured CD8^+^ T cells with ^15^N tracer-labeled NH_4_Cl in the presence or absence of 2-KG. The addition of 2-KG significantly increased the amount of ^15^N tracer-labeled glutamate, suggesting that 2-KG enhanced the assimilation of ammonia into glutamate in CD8^+^ T cells (Fig. 3j). Together, the results indicated that GOT1 was required for CD8^+^ T cells to generate 2-KG, which promoted the assimilation of free ammonia and cell survival.

## GOT1 promotes T_eff_ cell formation in acute LCMV infection

**Extended Data Fig. 5:**
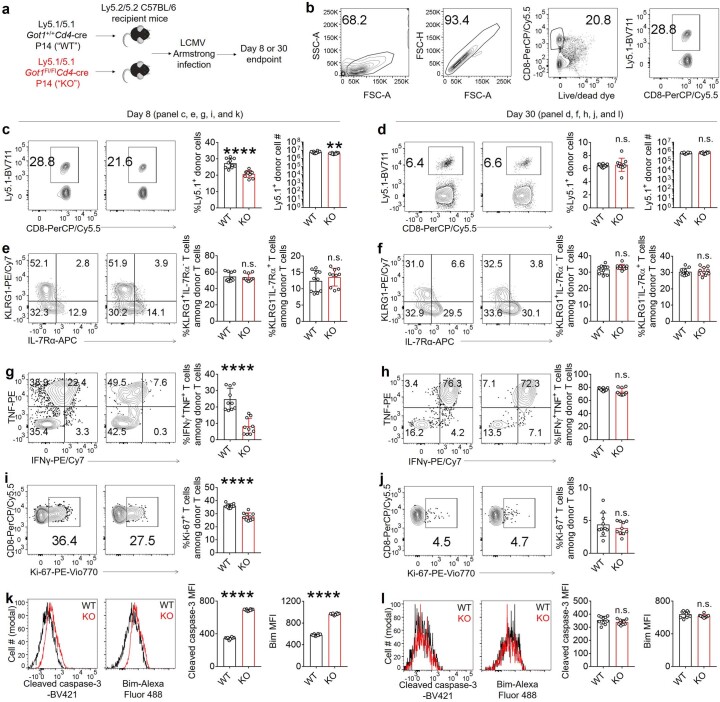
CD8^+^ T cell responses require GOT1 during acute infection. **a**, Experimental design illustration. **b**, FACS gating strategies used in **c**–**l**. **c**–**l**, Contour plots, histograms, and bar graphs show the flow cytometry staining results of Ly5.1^+^ donor P14 CD8^+^ T cells (**c**,**d**), KLRG1 and IL-7Rα (**e**,**f**), cytokines (**g**,**h**), Ki-67 (**i**,**j**), and cleaved caspase-3 and Bim (**k**–**l**) in *Got1*-deficient and sufficient P14 CD8^+^ T cells. Cells were stimulated with GP_33–41_ peptide before the flow cytometry staining (**g**,**h**). Data were pooled from two independent experiments (**c**–**l**) with ten C57BL/6 mice in each group receiving *Got1*-deficient and sufficient donor P14 CD8^+^ T cells. The results are presented as mean ± s.d. **P<0.01; ***P<0.001; ****P<0.0001; n.s., not significant. Comparisons were performed using a two-tailed Student’s *t*-test (**c**, **d**, %KLRG1^−^IL-7Ra^+^ T cells among donor T cells in **e**, **f**, **g**, **h**, **i**, **j**, **k**, **l**; data points were normally distributed) or the two-tailed Mann-Whitney test (%KLRG1^+^IL-7Ra^−^ T cells among donor T cells in **e**; data points were not normally distributed). *P* values in **c**: 1.6 × 10^−5^, 0.006; in **d**: 0.675, 0.745; in **e**: 0.752, 0.397; in **f**: 0.127, 0.738; in **g**: 6.4 × 10^−6^; in **h**: 0.069; in **i**: 2.4 × 10^−4^; in **j**: 0.3959; in **k**: 1.1 × 10^−5^, <1.0 × 10^−15^; in **l**: 0.194, 0.088. Six-week-old female mice were used (**b**–**l**). Source data

Because the expression levels of GOT1 transiently increased on day 8 after LCMV Armstrong infection (Fig. 1c), we investigated the potential role of GOT1 in regulating the formation of effector CD8^+^ T cells during LCMV Armstrong acute infections (Extended Data Fig. 5a). *Got1* deficiency modestly but significantly reduced the numbers of virus-specific CD8^+^ T cells on day 8 but not day 30 after LCMV Armstrong infection (Extended Data Fig. 5b–d). *Got1* deficiency did not influence the protein levels of *KLRG1* or IL-7Rα (Extended Data Fig. 5e–f). *Got1* deficiency decreased effector cytokine production and cell proliferation and increased the expression levels of apoptosis-related protein markers on day 8 but not on day 30 (Extended Data Fig. 5g–l). This selective requirement of GOT1 for the accumulation of CD8^+^ T_eff_ cells on day 8, but not for the accumulation of CD8^+^ T_mem_ cells on day 30, is consistent with the selective expression of GOT1 in CD8^+^ T_eff_ cells, but only at basal levels in CD8^+^ T_mem_ cells (Fig. 1c).

**Extended Data Fig. 6:**
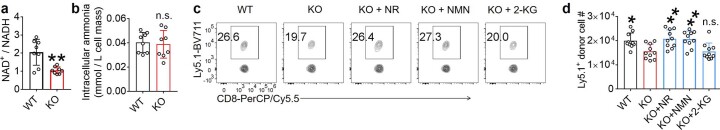
GOT1 maintains the NAD^+^/NADH ratio in acute LCMV infections. **a**,**b**, Bar graphs display NAD^+^/NADH ratios (**a**) and amounts of ammonia (**b**) in *Got1*-deficient and sufficient donor P14 CD8^+^ T cells isolated from C57BL/6 host mice infected 8 days earlier with LCMV Armstrong. **c**,**d**, Splenocytes were isolated from host mice 8 days after LCMV Armstrong infection and cultured with GP_33–41_ peptide in the presence or absence of the indicated compounds for 1 day. Flow cytometry contour plots (**c**) and a bar graph (**d**) show the percentages of *Got1*-deficient and sufficient Ly5.1^+^ donor T cells among CD8^+^ host T cells. Data are combined from two experiments with eight (**a**–**b**) or ten (**d**) mice in total. The data are presented as mean ± s.d. *P<0.05; **P<0.01; n.s., not significant. Comparisons were performed using a two-tailed Student’s *t*-test (**a** and **b**; data points were normally distributed) and one-way ANOVA (**d**). *P* values in **a**: 0.002; in **b**: 0.760; in **d**: 0.017, 0.004, 0.005, >0.9999. Six-week-old female mice were used (**a**–**d**). Source data

In acute LCMV Armstrong infections, *Got1* deficiency significantly reduced the NAD^+^/NADH ratio (Extended Data Fig. 6a) but did not influence the abundance of ammonia (Extended Data Fig. 6b). Supplementation with the NAD^+^ precursor molecules NR and NMN, but not the ammonia scavenger 2-KG, restored numbers of *Got1* KO CD8^+^ T cells (Extended Data Fig. 6c,d), suggesting that GOT1 promoted the formation of CD8^+^ T_eff_ cells in acute infections dependent on the traditional function of GOT1 in maintaining the NAD^+^/NADH ratio.

## *Got1-* deficient CD8^+^ T cells are similar to ammonia-treated WT CD8^+^ T cells

**Fig. 4:**
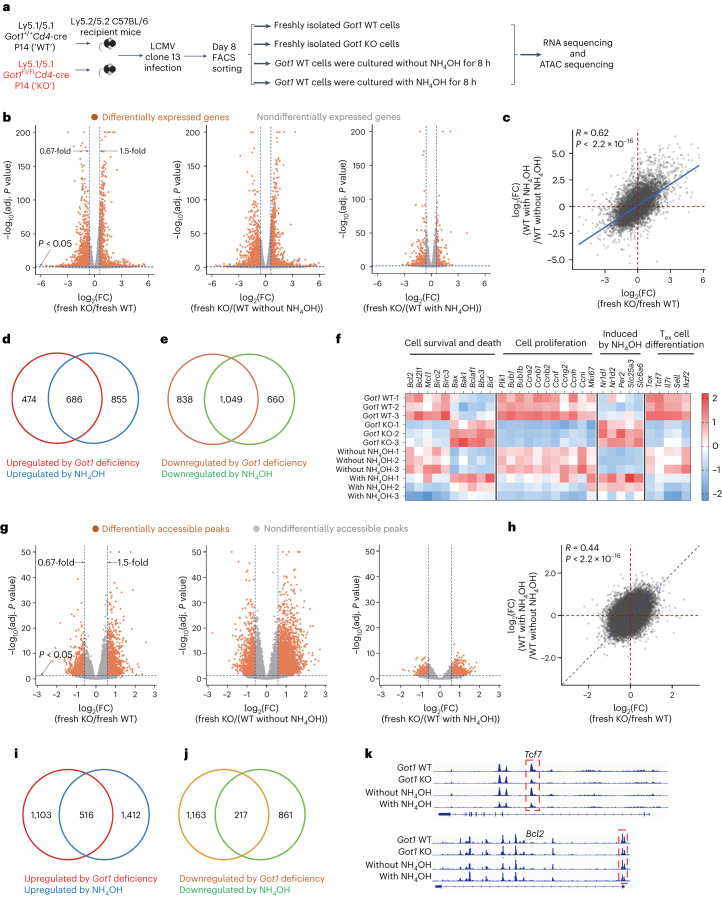
*Got1*-deficient CD8^+^ T cells are similar to ammonia-treated *Got1*-sufficient CD8^+^ T cells. **a**, Experimental design. *Got1*-deficient and sufficient donor P14 CD8^+^ T cells were isolated from C57BL/6 host mice infected with LCMV clone 13 8 d earlier. *Got1*-deficient P14 cells and one fraction of *Got1*-sufficient P14 cells were directly used for RNA-sequencing analysis and ATAC-sequencing analysis. The other fractions of *Got1*-sufficient P14 cells were cultured with anti-CD3 and anti-CD28 in the presence or absence of 1 mM ammonium hydroxide for 8 h before RNA-sequencing analysis and ATAC-sequencing analysis. **b**,**g**, Volcano plots show the differentially expressed genes (**b**) or differentially accessible peaks (**g**). **c**,**h**, Dot plots show the correlation between *Got1* deficiency-induced changes in gene expression and ammonia treatment-induced changes in gene expression (**c**) or the correlation between *Got1* deficiency-induced changes in chromatic openness and ammonia treatment-induced changes in chromatic openness (**h**) in the indicated groups of cells. **d**,**e**,**i**,**j**, Venn diagrams show the numbers of genes (**d**,**e**) or accessible peaks (**i**,**j**) overlapping between the indicated groups of comparisons. **f**, Heat map shows the mRNA expression *z* scores of the indicated genes in the four groups of cells. **k**, ATAC-sequencing signal profiles across *Tcf7* and *Bcl2* loci in the indicated groups of P14 CD8^+^ T cells. *n* = 3 mice in each of the four groups (**b**–**k**). Comparisons were performed with a two-sided Wald test, and *P* values were adjusted with the Benjamini–Hochberg procedure (**b**,**g**) or a two-tailed Student’s *t* test (**c**,**h**). Six-week-old male mice were used (**b**–**k**). FC, fold change. Source data

To compare the effect of *Got1* deficiency versus ammonia treatment on the global transcriptional profiles and epigenetic landscapes, we performed RNA sequencing and assay for transposase-accessible chromatin (ATAC) sequencing analyses of four groups of CD8^+^ T cells (Fig. 4a). The pair of *Got1* KO P14 CD8^+^ T cells and NH_4_OH-treated *Got1* WT P14 CD8^+^ T cells shared the highest degree of similarity with the least differentially expressed genes (Fig. 4b). We further correlated *Got1* deficiency-induced changes in gene expression with those caused by NH_4_OH treatment and found that these two groups of differentially expressed genes closely correlated with each other (*R* = 0.62) (Fig. 4c). 686 or 1,049 genes were unanimously increased or decreased, respectively, by *Got1* deficiency and by NH_4_OH treatment (Fig. 4d,e).

**Extended Data Fig. 7:**
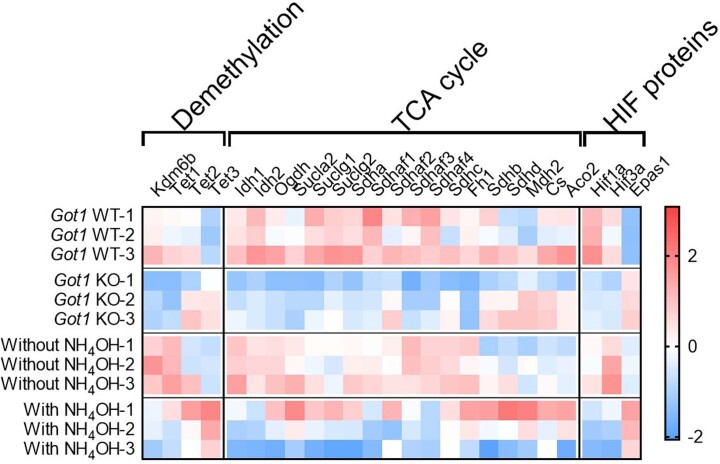
*Got1* and ammonia influence the expression of 2-KG-related genes. *Got1*-deficient and sufficient donor P14 CD8^+^ T cells were isolated from C57BL/6 host mice infected with LCMV clone 13 8 days earlier, similar to those described in Fig. 4. *Got1*-deficient P14 cells and *Got1*-sufficient P14 cells were used for RNA sequencing and ATAC sequencing analyses. The heat map shows the mRNA expression z-scores of the indicated genes in the four groups of cells. N = 3 mice in each of the four groups. Six-week-old male mice were used. Source data

Both *Got1* deficiency and NH_4_OH treatment decreased the expression of genes promoting cell survival and increased the expression of genes promoting cell death (Fig. 4f). *Got1* deficiency and ammonia treatment also decreased the expression levels of the cell proliferation-associated genes, with the exception of *Mki67*, which was decreased by *Got1* deficiency but not ammonia treatment. One possible explanation was that the 8 h ammonia treatment in vitro was not sufficiently long to markedly decrease the expression of *Mki67*. Multiple genes known to be induced by NH_4_OH, such as *Nr1d1, Nr1d2, Per2*, *Slc25a3* and *Slc6a6* (refs. ^31,32^), were also upregulated in *Got1* KO T cells, thereby suggesting that *Got1* deficiency conferred T cells a phenotype resembling that of NH_4_OH-treated T cells. Ammonia exposure also inhibited the expression of *Tox*, which is required for maintaining the phenotype and survival of T_ex_ cells^33–36^. Moreover, *Tcf7*, *Il7r*, *Sell* and *Ikzf2*, whose expression is decreased by *Tox* deficiency^33–36^, were also inhibited by both *Got1* deficiency and ammonia treatment (Fig. 4f). Similar to the gene expression, global chromatin accessibility was also influenced by *Got1* deficiency and by ammonia treatment, as demonstrated by volcano plots (Fig. 4g), correlation analysis (Fig. 4h) and Venn diagrams comparing the open chromatin regions between the indicated groups of CD8^+^ T cells (Fig. 4i,j). *Tcf7* and *Bcl2*, whose gene expression levels were unanimously decreased by *Got1* deficiency and ammonia treatment, were also less accessible in *Got1* KO T cells and in ammonia-treated T cells (Fig. 4k). Because 2-KG is involved in multiple biological processes, such as demethylation, the TCA cycle and HIF proteins, we conducted further analysis to examine whether *Got1* deficiency and ammonia treatment influenced the expression levels of genes involved in these biological processes (Extended Data Fig. 7). We found that both *Got1* deficiency and ammonia treatment decreased the expression levels of genes involved in demethylation, such as *Kdm6b* and *Tet1*. Conversely, the expression levels of *Tet3* were increased in *Got1*-deficient cells and ammonia-treated cells. This increase in *Tet3* expression may represent a compensatory mechanism to counterbalance the reduced expression of *Kdm6b* and *Tet1*. Furthermore, the expression levels of genes encoding TCA enzymes were generally decreased by *Got1* deficiency and ammonia treatment. Additionally, we found that *Got1* deficiency and ammonia treatment reduced the mRNA levels of *Hif1a* and *Hif3a* and increased the expression of *Epas*1 (encoding the HIF2a protein). Together, these results suggested that *Got1* deficiency influenced the transcriptional profiles and epigenetic landscapes of CD8^+^ T cells in a manner similar to ammonia treatment.

## GOT1 catalyzes an atypical chemical reaction in CD8^+^ T_ex_ cells

**Fig. 5:**
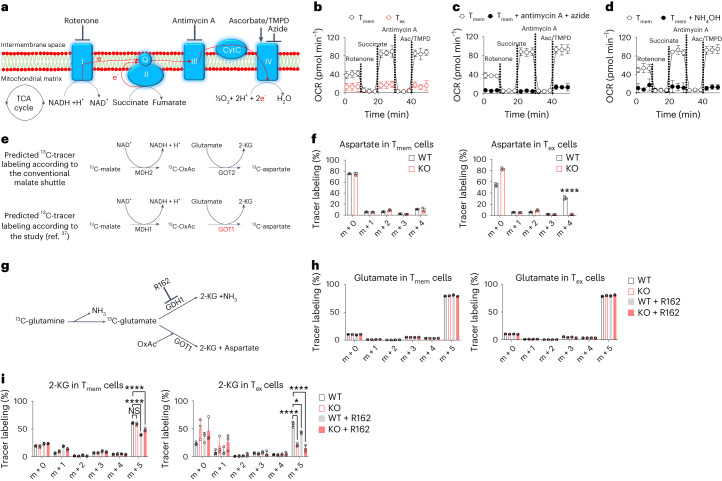
T_ex_ cells require GOT1 to produce 2-KG by an ‘ammonia-neutral pathway’. **a**, Electron flow through mitochondrial complexes. **b**–**d**, WT donor P14 CD8^+^ T cells were isolated from C57BL/6 host mice that were infected with LCMV clone 13 or LCMV Armstrong 30 d earlier. T_ex_ or T_mem_ donor P14 CD8^+^ cells were FACS-purified and permeabilized with PMP. Mitochondrial complex inhibitors (rotenone and antimycin A inhibit complexes I and III, respectively) and substrates (succinate, a substrate for complex II; Asc and TMPD, substrates for complex IV) were supplemented sequentially into the T cell culture as indicated. Line graphs show the OCR values of PMP-permeabilized T_ex_ cell versus T_mem_ cells (**b**), T_mem_ cell versus T_mem_ cells treated with antimycin A and azide (**c**), or T_mem_ cell versus T_mem_ cells treated with NH_4_OH (**d**) in Seahorse electron flow assays. **e**, Illustration of the incorporation of the tracer ^13^C from ^13^C-malate into ^13^C-aspartate. **f**, Bar graphs show amounts of ^13^C-aspartate in T_mem_ cells and T_ex_ cells. ‘m + 4’ indicates aspartate with four ^13^C atoms. **g**, Illustration of the incorporation of the tracer ^13^C from ^13^C-glutamine into ^13^C-glutamate or ^13^C-2-KG. **h**,**i**, Bar graphs show amounts of ^13^C-glutamate (**h**) or ^13^C-2-KG (**i**) in T_mem_ cells and T_ex_ cells. ‘m + 5’ indicates glutamate (**h**) or 2-KG (**i**) with five ^13^C atoms. Data are combined from two experiments with six mice (**b**–**d**), 15 host mice containing *Got1*-sufficient donor P14 CD8^+^ T cells, and 30 host mice containing *Got1*-deficient donor P14 CD8^+^ T cells (**f**,**h**,**i**). Data are mean ± s.d. *P < 0.05; ****P < 0.0001. Comparisons were performed with two-way ANOVA (**f**,**h**,**i**). In **f**, *P* = 2.0 × 10^−15^; in **i**, *P* = 0.066993749, *P* < 1.0 × 10^−15^, *P* = 1.0 × 10^−14^, *P* = 6.7 × 10^−7^, *P* = 0.025248392 and *P* = 2.2 × 10^−8^. Six-week-old female mice were used (**b**–**d**,**f**,**h**,**i**). Source data

To monitor electron transport in CD8^+^ T cells (Fig. 5a), we performed mitochondrial electron flow analysis of CD8^+^ T_ex_ cells and CD8^+^ T_mem_ cells by using a Seahorse extracellular flux analyzer (Fig. 5b–d). We pretreated CD8^+^ T_ex_ cells and CD8^+^ T_mem_ cells with plasma membrane permeabilizer (PMP), which permeabilizes plasma membranes but not mitochondrial membranes. Therefore, PMP treatment enables the monitoring of mitochondrial metabolism without the need for purifying mitochondria. The oxygen consumption rate (OCR) in CD8^+^ T_mem_ cells responded robustly to rotenone (an inhibitor of mitochondrial complex I), succinate (a substrate for mitochondrial complex II), antimycin A (an inhibitor of mitochondrial complex III), and ascorbate (Asc) and N,N,N′,N′-tetramethyl-para-phenylene-diamine (TMPD) (substrates for mitochondrial complex IV), suggesting that CD8^+^ T_mem_ cells underwent active electron transportation through the mitochondrial complex to oxygen. By contrast, the OCR was much lower in CD8^+^ T_ex_ cells, and CD8^+^ T_ex_ cells responded poorly to inhibitors and substrates of mitochondrial complexes (Fig. 5b). The OCR of CD8^+^ T_ex_ cells resembled that of CD8^+^ T_mem_ cells treated with antimycin A and azide (inhibitors of mitochondrial complex III and IV) or NH_4_OH, respectively (Fig. 5c,d). Collectively, these results suggested that electron transport through ETC of CD8^+^ T_ex_ cells was inhibited with respect to that in CD8^+^ T_mem_ cells.

GOT1 catalyzes an atypical chemical reaction that generates 2-KG and aspartate from oxaloacetate and glutamate in human Jurkat leukemic T cells when respiration is inhibited^37^. We examined whether GOT1 also catalyzed this atypical chemical reaction in CD8^+^ T_ex_ cells (Fig. 5e). We pulsed CD8^+^ T_ex_ cells with ^13^C tracer-labeled malate. In the conventional malate shuttle, GOT2, but not GOT1, is required to generate 2-KG and aspartate. *Got1* deficiency was not expected to affect the incorporation of ^13^C tracer into aspartate. If GOT1 catalyzed the atypical chemical reaction, as previously reported^37^, the incorporation of the ^13^C tracer into aspartate would be affected by *Got1* deficiency. *Got1* WT and *Got1* KO CD8^+^ T_mem_ cells generated comparable amounts of ^13^C tracer-labeled aspartate (Fig. 5f), thus suggesting that CD8^+^ T_mem_ cells underwent the conventional malate shuttle chemical reactions and did not require GOT1 to generate aspartate. By contrast, *Got1* KO CD8^+^ T_ex_ cells generated less ^13^C tracer-labeled aspartate than *Got1* WT CD8^+^ T_ex_ cells (Fig. 5f), suggesting that CD8^+^ T_ex_ cells required GOT1 to generate aspartate from malate. Collectively, these results suggested that GOT1 catalyzes an atypical chemical reaction in CD8^+^ T_ex_ cells with respiratory inhibition.

## CD8^+^ T_ex_ cells require GOT1 to catabolize glutamate

Ammonia is produced by the deamination of glutamine and glutamate (Fig. 5g). The initial step of glutaminolysis is the conversion of glutamine to glutamate and ammonia. After this initial step, two different chemical pathways catabolize glutamate. First, glutamate dehydrogenase (GDH)-1 converts glutamate to 2-KG and ammonia. Second, glutamate is converted to 2-KG by GOT1 through atypical chemical reactions, as illustrated above (Fig. 5e), or by GOT2 through the conventional malate shuttle. Because the ammonia scavenger 2-KG is generated without the production of free ammonia in the second pathway, we refer to this pathway as the ‘ammonia-neutral pathway’.

To test whether *Got1* deficiency influenced the production of free ammonia, we measured the rates of glutaminolysis by pulsing *Got1* WT or *Got1* KO CD8^+^ T_ex_ cells and CD8^+^ T_mem_ cells with ^13^C tracer-labeled glutamine. During the period of observation, *Got1* WT and *Got1* KO CD8^+^ T cells generated comparable amounts of ^13^C tracer-labeled glutamate from glutamine (Fig. 5h). These results suggested that *Got1* deficiency did not influence the initial deamination step of glutaminolysis, at least in the short term.

To examine the individual contributions of the GOT1-dependent ammonia-neutral pathway and GDH1-dependent ammonia-generating pathway in glutamine catabolism, we pulsed *Got1* WT and KO CD8^+^ T cells with ^13^C tracer-labeled glutamine in the presence or absence of GDH1 inhibitor R162 (Fig. 5g). Inhibiting GDH1 by R162 decreased the incorporation of ^13^C tracers into 2-KG in CD8^+^ T_mem_ cells, but the amounts of ^13^C tracers in 2-KG were comparable between *Got1* WT and KO CD8^+^ T_mem_ cells (Fig. 5i). These results suggested that glutamate relied on the GDH1-mediated pathway to generate 2-KG in CD8^+^ T_mem_ cells. By contrast, *Got1* deficiency decreased 2-KG production from glutamate in CD8^+^ T_ex_ cells. Furthermore, *Got1* deficiency combined with GDH1 inhibition almost completely blocked the conversion from glutamate to 2-KG (Fig. 5i). These results suggested that CD8^+^ T_mem_ cells and T_ex_ cells underwent the initial deamination step of glutaminolysis at comparable rates. At the second step, however, CD8^+^ T_ex_ cells had a greater reliance on the GOT1-mediated ammonia scavenger-generating pathway than CD8^+^ T_mem_ cells, which underwent GDH1-dependent deamination.

## 2-KG restores *Got1*-deficient CD8^+^ T cell-mediated antiviral responses

**Fig. 6:**
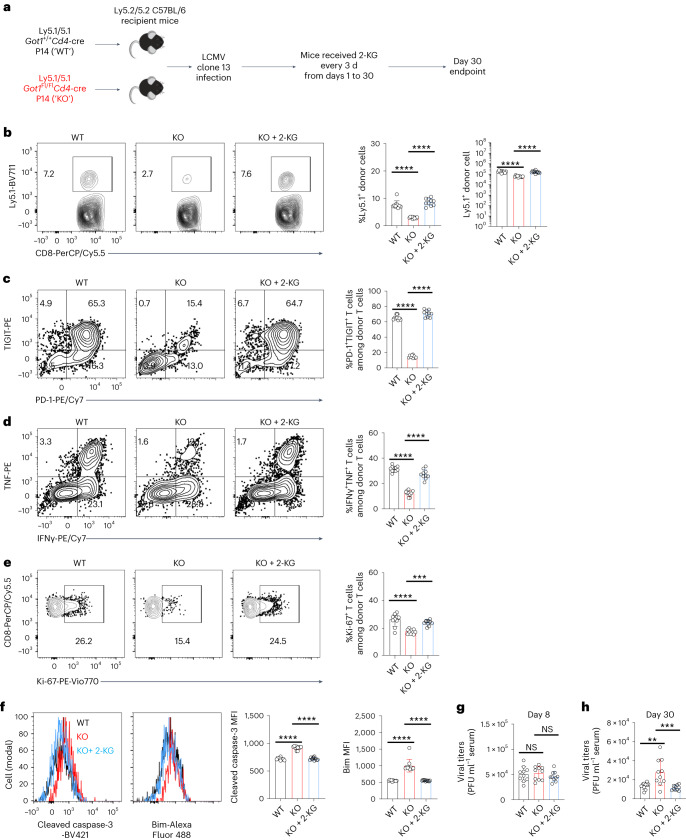
2-KG restores *Got1*-deficient CD8^+^ T cell antiviral responses in vivo. **a**, Experimental design. **b**–**f**, Contour plots, histograms and bar graphs show the flow cytometry staining results of Ly5.1^+^ donor P14 CD8^+^ T cells (**b**), PD-1 and TIGIT (**c**), cytokines (**d**), Ki-67 (**e**), and cleaved caspase-3 and Bim (**f**) in *Got1*-deficient and sufficient P14 CD8^+^ T cells. Cells were stimulated with GP_33–41_ peptides before flow cytometry staining (**d**). **g**,**h**, Bar graphs show the viral titers at day 8 (**g**) and day 30 (**h**) after infections. Data were pooled from two independent experiments (**b**–**h**). *n* = 10 C57BL/6 mice per group. The results are presented as mean ± s.d. **P < 0.01; ***P < 0.001; ****P < 0.0001. Comparisons were performed with one-way ANOVA. In **b**, *P* = 1.6 × 10^−9^, *P* = 3.2 × 10^−11^, *P* = 1.3 × 10^−7^ and *P* = 5.8 × 10^−7^; in **c**, both *P* = 1.1 × 10^−14^; in **d**, *P* = 4.5 × 10^−14^ and *P* = 1.1 × 10^−11^; in **e**, *P* = 2.5 × 10^−6^ and *P* = 0.00015972; in **f**, *P* = 1.7 × 10^−14^, *P* = 1.9 × 10^−14^, *P* = 3.7 × 10^−9^ and *P* = 4.0 × 10^−9^; in **g**, *P* = 0.937 and *P* = 0.477; in **h**, *P* = 0.002629596 and *P* = 0.000658958. Six-week-old female mice were used (**b**–**h**). Source data

To test whether 2-KG enhanced *Got1* KO CD8^+^ T cell-mediated antiviral responses in vivo, we adoptively transferred *Got1* KO and WT P14 CD8^+^ T cells into C57BL/6 mice and infected these mice with LCMV clone 13. We treated these mice with 2-KG or vehicle control (Fig. 6a). The total numbers of *Got1* KO donor T cells significantly increased after 2-KG treatment to a level comparable to that observed in *Got1* WT donor T cells (Fig. 6b). In addition, 2-KG significantly increased PD-1 expression (Fig. 6c) and promoted IFNγ and TNF production in *Got1* KO donor T cells (Fig. 6d). Furthermore, 2-KG restored the expression of Ki-67 (Fig. 6e) and decreased the expression levels of apoptosis-associated cleaved caspase-3 and Bim in *Got1* KO donor T cells (Fig. 6f). Viral titers were comparable between the ‘*Got1* WT’ group and the ‘*Got1* KO’ group on day 8 after LCMV clone 13 infections (Fig. 6g). However, on day 30, C57BL/6 mice that received *Got1* KO P14 CD8^+^ T cells exhibited higher viral titers than those receiving *Got1* WT P14 CD8^+^ T cells. Treatment with 2-KG reduced the viral titers in the serum of C57BL/6 mice that received *Got1* KO P14 CD8^+^ T cells (Fig. 6h). These results suggest that *Got1* deficiency affected antiviral CD8^+^ T cell responses, which was restored by 2-KG treatment.

## 2-KG decreases ammonia and restores survival of *GOT1*-deficient human CD8^+^ T cells

**Fig. 7:**
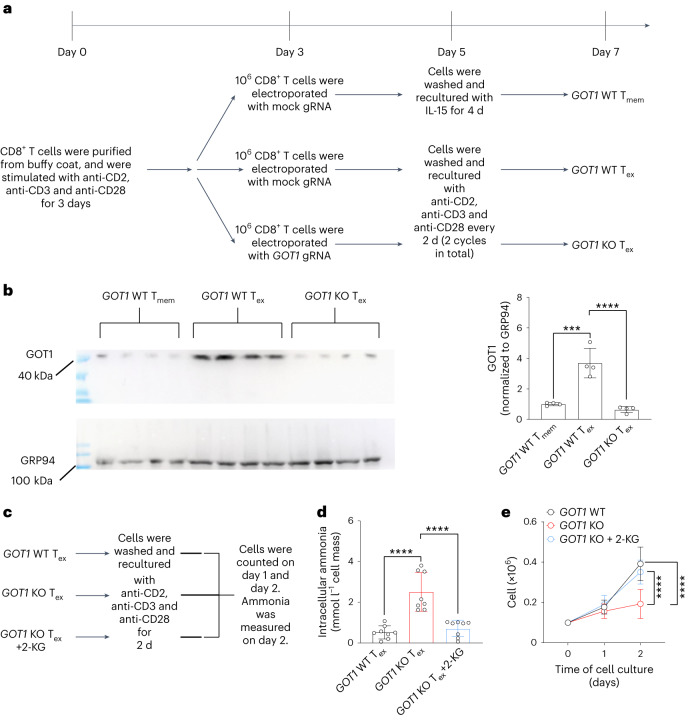
2-KG decreases ammonia and restores survival of *GOT1*-deficient human CD8^+^ T cells. **a**, Experimental design of **b**. Healthy donor CD8^+^ T cells were electroporated with Cas9 protein and *GOT1-*specific gRNA and were differentiated into T_ex_-like cells or T_mem_-like cells. **b**, Western blot images show the expression of GOT1 in the indicated populations of cells. GRP94 was used as a loading control. A bar graph shows the results of densitometric quantification of the GOT1 immunoblot bands. **c**, Illustration of the experimental design of **d** and **e**. **d**, Bar graph shows the concentrations of ammonia in CD8^+^ T cells. **e**, A line graph shows the numbers of cells on day 1 and day 2 after restimulation with anti-CD2, anti-CD3 and anti-CD28. Data are cumulative from two independent experiments with eight healthy donors in total. Results are mean ± s.d. ***P < 0.001; ****P < 0.0001. Comparisons were performed with one-way ANOVA (**b**,**d**) or two-way ANOVA (**e**). In **b**, *P* = 0.000192726 and *P* < 1.0 × 10^−15^; in **d**, both *P* < 1.0 × 10^−15^; in **e** (from top to bottom), *P* = 6.4 × 10^−11^ and *P* = 6.7 × 10^−8^. Source data

To examine the expression levels of GOT1 protein in human T_ex_ cells and T_mem_ cells, we followed a previously published protocol^38^ to generate T_ex_ cells and T_mem_ cells in vitro (Fig. 7a). T_ex_ cells expressed higher levels of GOT1 protein than T_mem_ cells (Fig. 7b). We used CRISPR/Cas9 technology to generate human *GOT1*-deficient CD8^+^ T cells, which expressed low levels of GOT1 protein (Fig. 7b). To examine whether *GOT1* deficiency increased ammonia accumulation in T_ex_ cells and caused *GOT1* KO CD8^+^ T cell death, we treated *GOT1* KO T_ex_ cells with 2-KG (Fig. 7c). *GOT1* KO T_ex_ cells produced higher levels of ammonia than *GOT1* WT T_ex_ cells, and 2-KG decreased the ammonia in *GOT1* KO T_ex_ cells to a level comparable to that in *GOT1* WT T_ex_ cells (Fig. 7d). Furthermore, 2-KG restored *GOT1* KO T_ex_ cell survival (Fig. 7e), in line with our mouse model results showing that 2-KG increased the numbers of T_ex_ cells during LCMV clone 13 chronic infection (Fig. 6b). Collectively, these results suggested that human T_ex_ cells relied on GOT1 to detoxify ammonia.

We discovered that CD8^+^ T_ex_ cells expressed high levels of GOT1 during chronic viral infection. GOT1 promoted the survival of human and mouse CD8^+^ T_ex_ cells by catalyzing an unconventional chemical reaction producing 2-KG. 2-KG assimilated ammonia and enabled sustainable ammonia-neutral glutaminolysis in CD8^+^ T_ex_ cells. Our work sheds new light on the plasticity of GOT1-catalyzed chemical reaction networks and reveals that T_ex_ cells rewire the malate shuttle-associated metabolic pathways when respiration is inhibited.

The longitudinal expression kinetics of GOT1 resembled that of the inhibitory receptors PD-1 and TIGIT, which were transiently expressed during acute infection and persistently expressed during chronic infection. *Got1* deficiency shrank the pool of CD8^+^ T cells expressing PD-1 and TIGIT but did not affect the homeostasis of naïve CD8^+^ T cells, suggesting that GOT1 was selectively required for the survival of PD-1^+^TIGIT^+^ CD8^+^ T cells. This finding is reminiscent of the requirement of TOX for maintaining the survival of CD8^+^ T_ex_ cells but not naïve T cells^33–36^. The selective expression of GOT1 in T_ex_ cells presents an opportunity to regulate the metabolism and survival of T_ex_ cells through pharmacological or genetic approaches.

Environmental ammonia’s toxicity has been extensively studied^39,40^. In contrast to environmental ammonia, whose exposure is rare, endogenous ammonia is constantly produced from amino acid metabolism and should be detoxified through continuously active mechanisms. The urea cycle is active in the liver and detoxifies ammonia by converting ammonia into urea, which is removed through excretion^41^. A recent study has suggested that the urea cycle is also active in T cells^42^. The current study suggested that the GOT1-mediated production of 2-KG assimilated ammonia and protected CD8^+^ T cells against high concentrations of free ammonia-induced cell death. This GOT1-mediated mechanism complements the urea cycle, thereby preventing the in situ accumulation of ammonia in T cells, and allowing antiviral T cells to undergo glutamine catabolism in a sustainable manner.

Our data revealed that GOT1 catalyzes an unconventional chemical reaction in CD8^+^ T_ex_ cells. This observation confirms previous reports showing that GOT1 is required to convert oxaloacetate and glutamate into aspartic acid and 2-KG when the respiratory chain is inhibited^37^. *Got1*-deficient and *Got1*-sufficient CD8^+^ T_ex_ cells had comparable levels of aspartic acid. One possible explanation is that the aspartic acid transporter in *Got1*-deficient CD8^+^ T_ex_ cells imported exogenous aspartic acid and compensated for the decreased synthesis of aspartic acid. The comparable levels of aspartic acid between *Got1*-deficient and *Got1*-sufficient T_ex_ cells also suggested that synthesizing aspartic acid was not the only driving force in GOT1’s catalysis of the unconventional chemical reaction. Ammonia accumulation also contributed to reversing the conventional chemical reaction to produce the ammonia scavenger 2-KG.

Overall, our findings revealed that GOT1 is induced by persistent TCR stimulation during chronic infection. GOT1 catalyzes an unconventional chemical reaction in CD8^+^ T_ex_ cells under respiratory inhibition, thereby producing the ammonia scavenger 2-KG, which detoxifies ammonia and is required for CD8^+^ T_ex_ cell survival. This study suggests that CD8^+^ T_ex_ cells adapt to persistent extracellular antigen stimulation by rewiring glutamine catabolism from the ammonia-producing pathway to the ammonia-neutral pathway, which promote CD8^+^ T_ex_ cell metabolic fitness.

## Methods

### Mice

Mice were maintained in the German Cancer Research Center (DKFZ), a specific pathogen-free facility. All studies were performed in accordance with DKFZ regulations with approval by the German regional council at the Regierungspräsidium Karlsruhe (G-232/16). The *Got1*^Flox/Flox^ mice, under the full name C57BL/6N-^Got1tm1c(EUCOMM)Hmgu^/H, were ordered from the MRC Harwell Institute, Oxfordshire, UK. Exon 2 of *Got1* is flanked by two LoxP sites and is excised after crossing with a Cre-expression mouse strain. *Cd4*-Cre mice^18,19^ and P14 mice^43^ were from The Jackson Laboratory and have been backcrossed to C57BL/6N background for more than ten generations. Mice were housed with a 12-h day/12-h night cycle in a controlled environment at 20–24 °C and 45–65% humidity and were fed a regular chow diet (Kliba Nafag, 3437) ad libitum. We used sex-matched and age-matched (6–7-week-old) mice for each individual experiment. In rare cases, mice with fighting wounds were excluded from the experimental analysis. The sample collection and processing were not performed in a blinded manner.

### Human samples

For immunofluorescence analysis, HIV patient tissue sections were provided by the tissue bank of the German Center for Infection Research (DZIF) in accordance with the regulations of the tissue bank and the approval of the ethics committee of Heidelberg University. HIV-positive samples were from two male donors (59-year-old and 62-year-old) and 1 female donor (34-year-old). HIV-negative samples were also from 2 male donors (65-year-old and 74-year-old) and 1 female donor (51-year-old). Buffy coat human peripheral blood mononuclear cell (PBMC) samples from healthy donors were provided by the blood bank of Mannheim. There were eight healthy donors in total (24-year-old female, 26-year-old female, 26-year-old male, 40-year-old female, 69-year-old male, 64-year-old male, 58-year-old female and 40-year-old male). Both the immunofluorescence evaluation of human tissue sections and the flow cytometry analysis of T cells from healthy donor PBMCs were conducted in accordance with the Declaration of Helsinki. Written informed consent was obtained from the patients before the analysis. No compensation was offered.

### Human T cell Cas9 and guide RNA electroporation

Healthy donor CD8^+^ T cells were purified from PBMCs with a CD8^+^ T cell isolation kit (Miltenyi, 130-096-495) and were pre-activated with anti-CD2, anti-CD3 and anti-CD28 for 3 d before electroporation. We used the predesigned Alt-R CRISPR–Cas9 *GOT1*-specfic CRISPR RNA (crRNA) from Integrated DNA Technologies (design Hs.Cas9.GOT1.1.AA, target sequence ACATTCGGTCCTATCGCTACTGG; design Hs.Cas9.GOT1.1.AB, target sequence ACCTCGGCAAAGACTGACGGAGG and design Hs.Cas9.GOT1.1.AC, target sequence ACGAGTATCTGCCAATCCTGGG). crRNA was annealed with *trans*-activating CRISPR RNA (tracrRNA; Integrated DNA Technologies, 1072534) to form gRNA. We electroporated human CD8^+^ T cells with Cas9 protein and gRNA according to a previously published protocol^44^. Briefly, 9 µl *GOT1*-specfic gRNA (3 μl design Hs.Cas9.GOT1.1.AA, 3 μl design Hs.Cas9.GOT1.1.AB and 3 μl design Hs.Cas9.GOT1.1.AC, 50 μM stock for each gRNA) and 6 µl Cas9 protein (5 μg ml^−1^ stock, Invitrogen; A36499) were incubated at room temperature for 10 min. tracrRNA was mixed with Cas9 protein as a negative control. A total of 10^7^ CD8^+^ T cells were resuspended in 100 μl P2 solution (Lonza, V4XP-2032) and gently mixed with the RNA–Cas9 protein complexes. The mixture was transferred to a nucleofection cuvette and electroporated with a 4D nucleofector (Lonza, core unit AAF-1002B, X unit AAF-1002X) with the EH100 electroporation program. Electroporated T cells were cultured in IL-7 (Miltenyi, 130-095-361) and allowed to recover overnight. Then T cells were repeatedly stimulated with anti-CD2, anti-CD3 and anti-CD28 for 4 d to differentiate T cells into T_ex_-like T cells. Another fraction of cells was washed and cultured with IL-15 (Miltenyi, 130-095-762) for 4 d to differentiate into T_mem_-like T cells.

### LCMV infection

C57BL/6 mice were intravenously injected with 5,000 (for subsequent LCMV Armstrong infection) or 500 (for subsequent LCMV clone 13 infection) *Got1*-deficient or -sufficient P14 TCR-transgenic CD8^+^ cells. These C57BL/6 mice containing P14 CD8^+^ T cells were infected with LCMV Armstrong (intraperitoneal injection, 2 × 10^5^ plaque-forming units (PFU) per mouse) or LCMV clone 13 (intravenous injection, 2 × 10^6^ PFU per mouse). These mice were killed at the indicated time points (day 8 or day 30 as specified in the figure legends). Spleens were collected for flow cytometry analysis.

### B16 melanoma cell implantation

B16 melanoma cells were maintained in DMEM supplemented with 10% FBS, penicillin and streptomycin. To maintain the expression of GP_33–41_ and OVA, we used G418 and blasticidin to supplement the B16-GP_33–41_ and B16-OVA melanoma cell cultures, respectively. B16-GP_33–41_ cells were provided by H. Pircher at the Max Planck Institute of Immunobiology and Epigenetics. B16 and B16-OVA cell lines were provided by R. Carretero in the DKFZ-Bayer Immunotherapeutic Lab. Before tumor cell implantation, we shaved the mice and subcutaneously injected B16 melanoma cells (2 × 10^5^ cells per mouse) into the flanks. We measured tumor sizes every 2–3 d with calipers.

### Mouse primary T cell culture

We cultured mouse splenocytes or purified T cells in a complete RPMI 1640 medium supplemented with 10% FBS, HEPES, 2-mercaptoethanol and nonessential amino acids. To culture P14 cells for measuring GOT1 protein levels by western blotting, we cultured *Got1*-deficient or -sufficient P14 splenocytes (1 × 10^6^ ml^−1^ complete medium per well in a 24-well plate) with the cognate GP_33–41_ peptide (10 ng ml^−1^; GenScript, RP20257) and IL-2 (10 ng ml^−1^; BioLegend, 575408) for the indicated times. NR (5 mM; Cayman Chemical, 23132), NMN (10 µM; Cayman Chemical, 16411), malic acid (0.5 mM; Sigma-Aldrich, PHR1273), the cell membrane-permeable dimethyl-2-KG (10 mM), GSK-J4 (1 µM; Sigma-Aldrich, SML0701), the cell membrane-permeable octyl-(R)-2 hydroxyglutarate 2-hydroxyglutaric acid (2-HG) (10 mM; Sigma-Aldrich, SML2200) and R162 (20 µM; Sigma-Aldrich, 5380980001) were added as indicated. In some experiments, where indicated, purified T cells were cultured with anti-CD3 and anti-CD28 or NH_4_OH (0.977 μM to 8 mM, as indicated in the figures; Santa Cruz, sc-214535).

### Staining of human tissue sections and microscopy

Paraffin-embedded slides were deparaffinized and rehydrated before epitope retrieval at 95 °C for 20 min. Slides were then rinsed in cold tap water. Next, slides were stained with anti-GOT1 in a moist chamber at 4 °C overnight. Slides were washed three times in PBS plus 0.1% Triton X-100 before being stained with 2 μg ml^−1^ Alexa Fluor 488-conjugated donkey anti-rabbit secondary antibody for 60 min at room temperature. Slides were washed and stained with Alexa Fluor 647-conjugated anti-CD8a for 60 min at room temperature. Sections were washed and covered with DAPI-containing anti-fade reagent and mounted with a coverslip. A confocal microscope (Zeiss LSM 710, ZEN Black Software) was used to photograph the sections.

### Flow cytometry

For surface antigen staining, Fc receptor blockers anti-CD16/CD32 were used to prevent nonspecific antibody binding. Cells were incubated in FACS buffer (PBS supplemented with 0.5% FCS) with the fluorescently conjugated antibodies for 30 min on ice. DAPI or a Live/DEAD Fixable Dead Cell Stain kit (Thermo Fisher) was used to exclude the dead cells. For intracellular cytokine staining, cells were fixed with the fixation buffer containing 4% paraformaldehyde (PFA; BioLegend) first, then permeabilized with eBioscience permeabilization buffer. For staining nuclear antigens, cells were fixed and permeabilized with the eBioscience Foxp3/transcription factor staining buffer set on ice for at least 30 min. Samples were washed and run on an LSR II or LSR Fortessa flow cytometer. We used FACS Diva Software (version 9, BD Biosciences) to collect FACS data, and analyzed data in FlowJo software (10.1r1).

### Antibodies

Following antibodies were obtained from BioLegend: Alexa Fluor 488-conjugated donkey anti-rabbit secondary antibody (406416, 1:2,000), Alexa Fluor 647-conjugated anti-CD8a (clone C8/144B, 372906, 1:200), BV421 anti-mouse CD8a (clone 53-6.7, 100738, 1:200), PE Donkey anti-rabbit IgG (polyclonal, 406421, 1:1,000), PerCP/Cyanine5.5 anti-mouse CD8a (clone 53-6.7, 100734, 1:200), BV711 anti-mouse CD45.1 (clone A20, 110739, 1:400), PE anti-mouse TIGIT (clone 1G99, 142104, 1:400), PE/Cyanine7 anti-mouse PD-1 (clone 29F.1A12, 135216, 1:400), PE anti-mouse TNF (clone MP6-XT22, 506306, 1:400), PE/Cyanine7 anti-mouse IFN-γ (clone XMG1.2, 505826, 1:400), BV421 donkey anti-rabbit IgG (polyclonal, 406410, 1:1,000), BV421 anti-mouse CD4 (clone GK1.5, 100438, 1:200), APC/Cyanine7 anti-mouse CD4 (clone GK1.5, 100414, 1:200), BV42 anti-CD44 (clone IM7, 103040, 1:400), PE/Cyanine7 anti-CD62L (clone MEL-14, 104418, 1:400), APC anti-CD25 (clone 3C7, 101910, 1:400), PE anti-IL-7Rα (clone A7R34, 135010, 1:400), anti-CD16/32 (clone 93, 101330, 1:100), anti-mouse CD3 (clone 17A2, 100238, 2 ug ml^−1^), anti-mouse CD28 (clone 37.51, 02116, 2 ug ml^−1^), anti-human CD3 (clone OKT3, 317326, 2 ug ml^−1^), anti-human CD2 (clone TS1/8, 309236, 2 ug ml^−1^) and anti-human CD28 (clone CD28.2, 302934, 2 ug ml^−1^). Following antibodies were obtained from Cell Signaling Technology: anti-GOT1 (clone E4A4O, 34423S, 1:500 for flow cytometry and tissue section stainings, 1:1,000 for immunoblotting), anti-GRP94 (clone D6X2Q, 20292, 1:500), anti-NFAT1 (clone D43B1, 5861, 1:200), anti-Eomes (polyclonal, 4540, 1:200), anti-Blimp1 (clone C14A4, 9115, 1:200), rabbit IgG (polyclonal, 2729, 1:200), Alexa Fluor 488-conjugated anti-Bim (clone C34C5, 94805, 1:400) and anti-Cleaved Caspase-3 (clone 5A1E, 9664, 1:400). Anti-TOX (polyclonal, ab155768, 1:200) is from Abcam. Anti-Ki-67 PE-Vio770 (clone REA183, 130-120-419, 1:400) is from Miltenyi.

### Immunoblotting

Cells were lysed with RIPA lysis buffer. Proteins were resolved with 15% SDS-PAGE (70 V for 30 min and then 60–90 min at 100 V, until the blue indicator ran to the edge of the gel). Proteins were subsequently transferred onto PVDF membranes (400 mA, 90 min). The membranes were blocked with 5% BSA in PBS supplemented with Tween-20 (PBST) for 1 h at room temperature, then incubated overnight at 4 °C with anti-GOT1 and anti-GRP94. The PVDF membrane was washed three times with PBST, and then incubated with HRP-conjugated secondary antibodies at room temperature for 1 h. The membrane was developed with the ECL method, and the data were collected with a Fusion system (FX6 Edge, Vilber). We quantified the band intensities in the NIH ImageJ (Version 1.53t) program.

### ATAC sequencing

A total of 100,000 viable cells were washed in PBS; subsequently, nuclei were isolated with the cold lysis buffer. Nuclei were resuspended in ATAC tagmentation master mix buffer and incubated at 500*g* for 30 min at 37 °C. Transposed chromatin was purified for subsequent library preparation. Sequencing was performed at the DKFZ Genomics and Proteomics Core Facility on the High Seq 2000 v4 Paired-End 125 bp platform. The DKFZ High Throughput Sequencing Unit prepared and sequenced the library (Illumina NovaSeq 6000 Paired-End Read 100 bp). Briefly, the ATAC-sequencing data were first subjected to adapter trimming and low-quality read filtering with flexbar (version 2.5)^45^ with the following parameters: -u 5 -m 26 -ae RIGHT -at 2 -ao 1. The trimmed reads were mapped to the mouse reference genome (mm10) with Bowtie 2 (version 2.4.2)^46^ with parameters -X 2000–mm. Reads that mapped to mitochondrial DNA or those with low mapping quality (<30) were excluded from downstream analysis. Duplicate reads due to PCR amplification of single DNA fragments during library preparation were identified with Picard (version 2.17.3; available at http://broadinstitute.github.io/picard) and thus were removed from the downstream analysis. MACS2 (version 2.2.7.1)^47^ was used for calling open chromatin regions. To identify peaks with differential accessibility, we counted the deduplicated reads overlapping with peaks. DESeq2 (version 1.30.1)^48^ was then used for statistical comparison, with a similar procedure regarding analyzing the RNA-seq data. Peaks with adjusted *P* values less than 0.05 and fold changes above 1.5 were considered the differentially accessible peaks. The ATAC sequencing data have been deposited in the Genome Expression Omnibus database under accession number GSE220876.

### RNA sequencing

The DKFZ High Throughput Sequencing Unit prepared and sequenced the library (Illumina NovaSeq 6000 Paired-End Read 50 bp). We analyzed the sequencing data according to a previously described protocol^49^. Briefly, the RNA-sequencing reads were first subjected to adapter trimming and low-quality read filtering with flexbar (version 2.5)^45^ with the following parameters: -u 6 -m 36 -ae RIGHT -at 2 -ao 2. Reads that were mapped to the reference sequences of rRNA, tRNA, snRNA, snoRNA and miscRNA (available from Ensembl and RepeatMasker annotation) with Bowtie 2 (version 2.4.2)^46^ with default parameters (in --end-to-end & --sensitive mode) were excluded. The remaining reads were then mapped to the mouse reference genome (mm10) with STAR (version 2.7.7a)^50^ with key parameters --outFilterMismatchNmax 8 --outFilterMismatchNoverLmax 0.1 -- alignIntronMin 20 --alignIntronMax 1000000 --outFilterType BySJout --outFilterIntronMotifs RemoveNoncanonicalUnannotated. Reads that mapped to multiple genomic sites were discarded in the following analysis. HTSeq-count (version 2.0.1)^51^ was used to count reads mapped to annotated genes, with parameters -f bam -r pos -s no -a 10. Differentially expressed gene analysis was performed with the R package DESeq2 (version 1.30.1)^48^. In brief, size factor estimation was first conducted to normalize the data across samples, and this was followed by dispersion estimation to account for the negative binomial distributed count data in RNA sequencing. Finally, gene expression fold changes were calculated, and the significance of the gene expression difference was estimated with the Wald test. To control for the false discovery rate in multiple testing, the raw *P* values were adjusted with the Benjamini–Hochberg procedure. Genes with adjusted *P* values less than 0.05 and fold changes above 1.5 were considered differentially expressed. The RNA-sequencing data have been deposited in the Genome Expression Omnibus database under accession number GSE220876.

### ChIP–PCR

We crosslinked DNA and proteins (1% formaldehyde, 12 min), lyzed cells, collected nuclei and resuspended the pellets in 300 μl SDS lysis buffer (1% SDS, 10 mM EDTA, 50 mM Tris–HCl and protease inhibitors). Cells were sonicated (Covaris M220 sonicator, duty factor 15%, peak incident 75 W, 200 cycles per burst, 10 min) to shear chromatin before centrifugation. The supernatants were incubated with antibodies or IgG isotype control and incubated at 4 °C overnight. After that, 50 μl BSA-blocked Dynabeads Protein A/G were incubated with the supernatant at 4 °C overnight. The magnetic beads were washed before the DNA–protein complexes were eluted at (65 °C for 30 min). We then treated the eluted complexes using RNase (10 μg ml^−1^) and proteinase K (200 μg ml^−1^) to remove RNA and protein before recovering DNA using the Qiagen PCR purification kit. In the subsequent qPCR analysis (ABI Prism 7500 sequence detection system, Applied Biosystems), we used 3 μl eluted DNA, 5 μl SyberGreen master mixture and 2 μl primers (Source data for Extended Table 1) for each reaction. The abundance of DNA was calculated using the ΔCt values between immunoprecipitated or input samples.

### Quantification of NAD^+^ and NADH

NAD^+^ and NADH were determined with an NAD/NADH-Glo Assay kit (Promega, G9071) according to the manufacturer’s instructions. Briefly, 10^6^ cells were lysed with 30 µl lysis buffer and then split into two fractions. For measurement of NAD^+^, 15 µl lysate was mixed with 7.5 µl of 0.4 M HCl, incubated at 60 °C for 15 min, and neutralized with 7.5 µl Tris-base. To measure NADH, samples were incubated for 15 min at 60 °C before 15 µl Tris–HCl was added. Subsequently, equal amounts of a luciferin detection reagent were added before the luciferase signal was measured with a luminescence detector. NAD^+^ and NADH concentrations were calculated with standard curves.

### Quantification of the malate shuttle-associated metabolites

To measure the malate shuttle-associated metabolites, we adapted a previously published method^52^. In brief, 10^6^ cells were extracted in 100 µl ice-cold methanol with sonication on ice. For derivatization, 50 µl extract was mixed with 25 µl 140 mM 3-nitrophenylhydrazine hydrochloride, 25 µl methanol and 100 µl 50 mM ethyl-3-(3-dimethylaminopropyl) carbodiimide hydrochloride, and incubated for 20 min at 60 °C. Samples were separated by reversed-phase chromatography on an Acquity H-class UPLC system coupled to a QDa mass detector (Waters) with an Acquity HSS T3 column (100 mm × 2.1 mm, 1.8 µm, Waters), which was heated to 40 °C. Separation of derivatives was achieved by increasing the concentration of 0.1% formic acid in acetonitrile (B) in 0.1% formic acid in water (A) at 550 µl min^−1^ as follows: 2 min 15% B, 2.01 min 31% B, 5 min 54% B, 5.01 min 90% B, hold for 2 min and return to 15% B in 2 min. Mass signals for the following compounds were detected in single ion record mode by using negative detector polarity and 0.8 kV capillary voltage: malate (403.3 *m*/*z*; 25 V CV), succinate (387.3 *m*/*z*; 25 CV), fumarate (385.3 *m*/*z*; 30 V), citrate (443.3 *m*/*z*; 10 V), pyruvate (357.3 *m*/*z*; 15 V) and α-ketoglutarate (550.2 *m*/*z*; 25 CV). Data acquisition and processing were performed with the Empower3 software suite (Waters).

### Quantification of ammonia in CD8^+^ T cells

We used an Ammonia Assay Kit (Sigma-Aldrich, AA0100). We centrifuged cells (750*g*, 5 min, 4 °C), and lysed cells in 0.5% Triton X-100 on ice for 10 min. Subsequently, 20 µl lysate was mixed with 200 µl ammonia assay reagent and incubated at room temperature for 5 min. We then measured the absorbance at 340 nm with a spectrophotometer. We subsequently added 2 μl of L-GDH solution (in the Ammonia Assay Kit) to each well, gently mixed each well and incubated for 5 min at room temperature. The absorbance of each solution at 340 nm was again determined with a spectrophotometer. We obtained the ∆A340 value, and further calculated the amount of ammonia according to the instructions of the kit. Cell mass was quantified with graduated packed cell volume tubes (TPP Techno Plastic Products AG; Trasadingen, 870005). We divided the total amount of ammonia by the CD8^+^ T cell mass to calculate the concentrations of ammonia in cells.

### Metabolic tracer labeling

T cells were FACS-purified, pelleted and resuspended in PBS containing 1% FBS (1 × 10^6^ cells per ml, 0.5 ml). Tracers, including 2 mM ^13^C_5_-glutamine (Sigma-Aldrich, 605166), 1 mM ^15^NH_4_Cl (Sigma-Aldrich, 299251) and 0.5 mM ^13^C_4_-malic acid (Sigma-Aldrich, 750484), were added individually to the cells. Subsequently, 20 µM GDH1 inhibitor R162 (Calbiochem, 538098) was added as indicated. After 2 h, the labeling was quenched with cold 0.9% NaCl, and the tracers were washed out. The cell pellets were submitted to the Heidelberg Center for Organismal Studies and subjected for general tracing analysis to gas chromatography/mass spectrometry (GC/MS) and for glutamine, glutamate and alanine tracing analysis to liquid chromatography coupled to Ion Mobility Separation with Quadrupole Time of Flight (LC-IMS QTOF). For GC/MS analysis, frozen pellets of 10^6^ cells were extracted with 190 µl of 100% methanol (15 min, 70 °C). Each sample was mixed with 100 µl chloroform and shaken at 37 °C for 5 min. Then 200 µl water was added, and the samples were centrifuged (10 min, 11,000*g*) to separate polar and organic phases. The upper polar phase (300 µl) was transferred to a fresh tube before being dried in a vacuum concentrator. Sequential online methoximation and silylation reactions were performed using a MPS autosampler (Gerstel). Methoximation was performed by adding 20 µL 20 mg ml^−1^ methoxyamine hydrochloride (Sigma-Aldrich, 226904) in pyridine (Sigma-Aldrich, 270970) and incubation at 37 °C for 90 min in a Gerstel MPS Agitator Unit. For silylation reactions, 45 µl of N-methyl-N-(trimethylsilyl)trifluoroacetamide (MSTFA; Sigma-Aldrich, 69479) was added and samples were incubated at 37 °C for 30 min with gentle shaking. Before injection, samples were incubated at RT for 45 min. For GC/MS analysis, a GC-ToF system was used consisting of an Agilent 7890 Gas Chromatograph (Agilent) fitted with a Rxi-5Sil MS column (30 m × 0.25 mm × 0.25 µm; Restek Corporation) coupled to a Pegasus BT Mass Spectrometer (LECO Corporation). The GC was operated with an injection temperature of 250 °C and 1 µl sample was injected in splitless mode. The GC temperature program started with a 1 min hold at 40 °C followed by a 6 °C min^−1^ ramp up to 210 °C, a 20 °C min^−1^ ramp up to 330 °C and a bake-out at 330 °C for 5 min using Helium as carrier gas with constant linear velocity. The ToF MS was operated with ion source and interface temperature of 250 °C, a solvent cut time of 12 min and a scan range (*m*/*z*) of 50–600 with an acquisition rate of 17 spectra per second. Mass isotopologue distribution (MID) was determined using the DExSI software (version 1.11)^53^.

Determination of ^15^N tracer incorporation was done similarly as described^54^. Alanine, glutamate and glutamine content was analyzed after specific labeling with the fluorescence dye AccQ-Tag (Waters) according to the manufacturer’s protocol using an Acquity I-class UPLC system coupled to a VION Ion Mobility Separation QTof (Waters). Separation was carried out using a Cortecs C18 column (100 mm × 2.1 mm, 1.6 µm, Waters) at 40 °C. The mobile UPLC phase consisted of binary gradients of ACN with 0.1% formic acid (B) and 0.1% aqueous formic acid (A), flowing at 0.5 ml min^−1^. Analytes were initially eluted with 98% A and A was decreased linearly to 76% over 7.90 min. After this, the column was washed with 90% B for 1.49 min and re-equilibrated under the initial conditions for 2.9 min. Measurements were performed with an ESI source operated in positive mode (1.00 kV capillary voltage; source temperature 120 °C, desolvation temperature 550 °C; sample cone voltage 20 V; source offset voltage 50 V; observed *m*/*z* 150–700Da with a scan time of 0.300 s). Unifi software (Waters) was used to control the instrument and to acquire and process the MS data.

### Seahorse extracellular flux analysis

The Seahorse sensor cartridges were hydrated overnight at 37 °C before the assay, according to the manufacturer’s instructions. The culture plates were coated with poly d-lysine at 4 °C. To perform electron flow assay, we seeded T cells in mitochondrial assay solution (0.15 × 10^6^ cells per well) supplemented with 4 mM ADP, 2 µM FCCP, 10 mM sodium pyruvate, 2 mM malic acid and 1 nM PMP. The following compounds were injected into the culture plate sequentially: rotenone (2 µM), sodium succinate (10 mM), antimycin A (4 µM) and a mixture of 10 mM Asc and 100 µM TMPD. OCR values were recorded automatically with a Seahorse flux analyzer.

### Statistical analysis

No statistical methods were used to predetermine sample sizes but our sample sizes are similar to those reported in previous publications^2,55^. We used GraphPad Prism (v7.0.3) to perform statistical analysis. When comparing two groups, we first determined whether the data points were normally distributed. Statistical analysis of normally distributed data was performed with two-tailed Student’s *t* tests. Statistical analysis of data points that were not normally distributed was performed with two-tailed Mann–Whitney *U* tests (also known as the Wilcoxon rank sum test). Simultaneous comparisons of more than two groups were performed with one-way or two-way analysis of variance, as indicated in the figure legends. In all cases, *P* < 0.05 was considered statistically significant. Sample sizes are indicated in the figure legends. Data are presented as mean ± s.d., as specified in the figure legends. Data collection and analysis were not performed blind to the conditions of the experiments. We did not use a randomization protocol and assigned mice to experimental groups according to genotypes.

### Reporting summary

Further information on research design is available in the Nature Portfolio Reporting Summary linked to this article.

## Online content

Any methods, additional references, Nature Portfolio reporting summaries, source data, extended data, supplementary information, acknowledgements, peer review information; details of author contributions and competing interests; and statements of data and code availability are available at 10.1038/s41590-023-01636-5.

### Supplementary information

Reporting Summary

### Source data

Source Data Fig. 1 Statistical source data.

Source Data Fig. 2 Statistical source data.

Source Data Fig. 3 Statistical source data.

Source Data Fig. 4 Statistical source data.

Source Data Fig. 5 Statistical source data.

Source Data Fig. 6 Statistical source data.

Source Data Fig. 7 Statistical source data.

Source Data Fig. 8 Unprocessed western blots.

Source Data Extended Data Fig. 1 Statistical source data.

Source Data Extended Data Fig. 2 Statistical source data.

Source Data Extended Data Fig. 3 Statistical source data.

Source Data Extended Data Fig. 4 Statistical source data.

Source Data Extended Data Fig. 5 Statistical source data.

Source Data Extended Data Fig. 6 Statistical source data.

Source Data Extended Data Fig. 7 Statistical source data.

Source Data Extended Data Fig. 8 Primer sequences.

## Data Availability

RNA- and ATAC-sequencing data have been deposited in the GEO database under the accession code GSE220876. All other data are present in the manuscript and the Supplementary Information or from the corresponding authors upon reasonable request. Source data are provided with this paper.
