# Supplementary material for: The malate shuttle detoxifies ammonia in exhausted T cells by producing 2-ketoglutarate
Source: Nat Immunol. 2023 Oct 9;24(11):1921–32. doi: 10.1038/s41590-023-01636-5 (PMC10602850; doi:10.1038/s41590-023-01636-5)
Supplement: Supplementary file 1 — Reporting Summary [file 41590_2023_1636_MOESM1_ESM.pdf]

nature portfolio

Corresponding author(s): Guoliang Cui

Last updated by author(s): Aug 24, 2023

# Reporting Summary

Nature Portfolio wishes to improve the reproducibility of the work that we publish. This form provides structure for consistency and transparency in reporting. For further information on Nature Portfolio policies, see our Editorial Policies and the Editorial Policy Checklist.

## Statistics

For all statistical analyses, confirm that the following items are present in the figure legend, table legend, main text, or Methods section.

| n/a | Confirmed | |
|---|---|---|
| ☐ | ☒ | The exact sample size ($n$) for each experimental group/condition, given as a discrete number and unit of measurement |
| ☐ | ☒ | A statement on whether measurements were taken from distinct samples or whether the same sample was measured repeatedly |
| ☐ | ☒ | The statistical test(s) used AND whether they are one- or two-sided<br>*Only common tests should be described solely by name; describe more complex techniques in the Methods section.* |
| ☒ | ☐ | A description of all covariates tested |
| ☐ | ☒ | A description of any assumptions or corrections, such as tests of normality and adjustment for multiple comparisons |
| ☐ | ☒ | A full description of the statistical parameters including central tendency (e.g. means) or other basic estimates (e.g. regression coefficient) AND variation (e.g. standard deviation) or associated estimates of uncertainty (e.g. confidence intervals) |
| ☐ | ☒ | For null hypothesis testing, the test statistic (e.g. $F$, $t$, $r$) with confidence intervals, effect sizes, degrees of freedom and $P$ value noted<br>*Give P values as exact values whenever suitable.* |
| ☒ | ☐ | For Bayesian analysis, information on the choice of priors and Markov chain Monte Carlo settings |
| ☒ | ☐ | For hierarchical and complex designs, identification of the appropriate level for tests and full reporting of outcomes |
| ☒ | ☐ | Estimates of effect sizes (e.g. Cohen's $d$, Pearson's $r$), indicating how they were calculated |

*Our web collection on statistics for biologists contains articles on many of the points above.*

## Software and code

Policy information about availability of computer code

**Data collection**

We used FACS Diva Software (version 9, BD Biosciences) to collect FACS data.
FACS data were analyzed using FlowJo software (10.1r1).
Western blot data were collected by Fusion (FX6 Edge, Vilber).
We used the ABI Prism 7500 sequence detection system (SDS Software v1.2.3, Applied Biosystems) to collect the qPCR data.
We quantified the band intensities in the NIH ImageJ program (Version 1.53t).
Mass isotopologue distribution (MID) was determined using the DExSI software (Version 1.11).

**Data analysis**

FACS data were analyzed using Flowjo software (version 10.lrl). Western blot data were analyzed using Fusion FX6 Edge.
RNA sequencing reads were first subjected to adapter trimming and low-quality read filtering with flexbar (version 2.5) with the following parameters: -u 6 -m 36 -ae RIGHT -at 2 -ao 2. Reads that were mapped to the reference sequences of rRNA, tRNA, snRNA, snoRNA, and miscRNA (available from Ensembl and RepeatMasker annotation) with Bowtie 2 (version 2.4.2) with default parameters (in --end-to-end &--sensitive mode) were excluded. The remaining reads were then mapped to the mouse reference genome (mml0) with STAR (version 2.7.7a) with key parameters --outFilterMismatchNmax 8--outFilterMismatchNoverlmax 0.1-- alignlntronMin 20--alignlntronMax 1000000--outFilterType BySJout --outFilterlntronMotifs RemoveNoncanonicalUnannotated. Reads that mapped to multiple genomic sites were discarded in the following analysis. HTSeq-count (version 2.0.1) was used to count reads mapped to annotated genes, with parameters -f barn -r pos -s no -a 10. Differentially expressed gene analysis was performed with the R package DESeq2 (version 1.30.1). In brief, size factor estimation was first conducted to normalize the data across samples, and this was followed by dispersion estimation to account for the negative binomial distributed count data in RNA sequencing. Finally, gene expression fold changes were calculated, and the significance of the gene expression difference was estimated with the Wald test. To control for the false discovery rate in multiple testing, the raw p-values were adjusted with the Benjamini-Hochberg procedure.

ATAC sequencing data were first subjected to adapter trimming and low-quality read filtering with flexbar (version 2.5) with the following parameters: -u 5 -m 26 -ae RIGHT -at 2 -ao 1. The trimmed reads were mapped to the mouse reference genome (mmlO) with Bowtie 2 (version 2.4.2) with parameters -X 2000 --mm. Reads that mapped to mitochondrial DNA or those with low mapping quality(< 30) were excluded from downstream analysis. Duplicate reads due to PCR amplification of single DNA fragments during library preparation were identified with Picard (version 2.17.3; available at http://broadinstitute.github.io/picard) and thus were removed from the downstream analysis. MACS2 (version 2.2.7.1) was used for calling open chromatin regions. To identify peaks with differential accessibility, we counted the deduplicated reads overlapping with peaks. DESeq2 (version 1.30.1),

For manuscripts utilizing custom algorithms or software that are central to the research but not yet described in published literature, software must be made available to editors and reviewers. We strongly encourage code deposition in a community repository (e.g. GitHub). See the Nature Portfolio guidelines for submitting code & software for further information.

## Data

Policy information about availability of data

All manuscripts must include a data availability statement. This statement should provide the following information, where applicable:

- Accession codes, unique identifiers, or web links for publicly available datasets
- A description of any restrictions on data availability
- For clinical datasets or third party data, please ensure that the statement adheres to our policy

The GEO accession number for the RNA sequencing data and ATAC sequencing data is GSE220876. The dataset will become public from September 1, 2023. All data needed to evaluate the conclusions in the paper are present in the manuscript and the Supplementary Information. There are no data restrictions.

## Research involving human participants, their data, or biological material

Policy information about studies with human participants or human data. See also policy information about sex, gender (identity/presentation), and sexual orientation and race, ethnicity and racism.

| | |
|---|---|
| Reporting on sex and gender | For the in vitro culture studies: buffy coat human peripheral blood mononuclear cell (PBMC) samples from healthy donors were provided by the blood bank of Mannheim. There were 4 male donors and 4 female donors. We did not observe different phenotypes between the male and female donor T cells.<br><br>For the HIV patient tissue section studies: lymph node sections were provided by the tissue bank of the German Center for Infection Research. HIV-positive samples were from 2 male donors (59-year-old, 62-year-old) and 1 female donor (34-year-old). HIV-negative samples were also from 2 male donors (65-year-old, 74-year-old) and 1 female donor (51-year-old). |
| Reporting on race, ethnicity, or other socially relevant groupings | We did not consider the information of race, ethnicity, or other socially relevant groupings when we requested samples from the blood bank of Mannheim or from the tissue bank of the German Center for Infection Research (DZIF, Heidelberg, Germany). We also do not have such information. |
| Population characteristics | For the in vitro culture studies: we used PBMC from healthy volunteers from 24 to 69 years of age.<br>For the HIV patient tissue section studies: lymph node sections were provided by the tissue bank of the German Center for Infection Research. Donors were 34 to 74 years of age. |
| Recruitment | For the in vitro culture studies: Buffy coat PBMC samples from healthy donors were provided by the blood bank of Mannheim, Germany. When choosing samples, we have considered both age (from 18 to 70 years of age) and gender (both male and female), but we did not consider other factors, such as race, ethnicity, or other socially relevant groupings.<br><br>For the HIV patient tissue section studies: lymph node sections were provided by the tissue bank of the German Center for Infection Research (DZIF, Heidelberg, Germany). Samples were chosen based on HIV positive or negative irrespective of age, gender, ethnicity or any other bias that could influence study outcomes. |
| Ethics oversight | This study was performed in accordance with the approval of the ethics committee of Heidelberg University. |

Note that full information on the approval of the study protocol must also be provided in the manuscript.

# Field-specific reporting

Please select the one below that is the best fit for your research. If you are not sure, read the appropriate sections before making your selection.

☒ Life sciences ☐ Behavioural & social sciences ☐ Ecological, evolutionary & environmental sciences

For a reference copy of the document with all sections, see nature.com/documents/nr-reporting-summary-flat.pdf

# Life sciences study design

All studies must disclose on these points even when the disclosure is negative.

| | |
|---|---|
| Sample size | No formal statistical methods were used to predetermine sample sizes but our sample sizes are similar to those reported in previous publications |

| | |
|---|---|
| Data exclusions | We did not exclude data. |
| Replication | Experiments were repeated twice or three times, as indicated in the figure legends. |
| Randomization | For the comparison between wildtype and knockout mice, littermate mice were allocated into 2 groups based on genotypes (namely wildtype and knockout mice).<br>For the P14 T cell adoptive transfer experiments, C57Bl/6N mice were randomly allocated into different groups.<br>We did not use a randomization protocol for the rest of the experiments. |
| Blinding | Data collection and analysis were not performed blind to the conditions of the experiments, because investigators who planned the experiments also performed them. |

# Reporting for specific materials, systems and methods

We require information from authors about some types of materials, experimental systems and methods used in many studies. Here, indicate whether each material, system or method listed is relevant to your study. If you are not sure if a list item applies to your research, read the appropriate section before selecting a response.

## Materials & experimental systems

| n/a | Involved in the study |
|---|---|
| ☐ | ☒ Antibodies |
| ☒ | ☐ Eukaryotic cell lines |
| ☒ | ☐ Palaeontology and archaeology |
| ☐ | ☒ Animals and other organisms |
| ☒ | ☐ Clinical data |
| ☒ | ☐ Dual use research of concern |
| ☒ | ☐ Plants |

## Methods

| n/a | Involved in the study |
|---|---|
| ☒ | ☐ ChIP-seq |
| ☐ | ☒ Flow cytometry |
| ☒ | ☐ MRI-based neuroimaging |

## Antibodies

Antibodies used

Anti-GOT1 (clone E4A4O), Cell Signaling Technology, Cat# 34423S, dilution: 1:500 for flow cytometry and tissue section stainings; 1:1000 for immunoblotting
Alexa Fluor 488-conjugated donkey anti-rabbit secondary antibody, BioLegend, Cat# 406416, dilution: 1:2000
Alexa Fluor 647-conjugated anti-CD8a (clone C8/144B), BioLegend, Cat# 372906, dilution: 1:200
Anti-GRP94 (clone D6X2Q), Cell Signaling Technology, Cat# 20292, dilution: 1:500 for immunoblotting
Anti-NFAT1 (clone D43B1), Cell Signaling Technology, Cat# 5861, dilution: 1:200 for ChIP
Anti-TOX, polyclonal, Abcam, Cat# ab155768, dilution: 1:200 for ChIP
Anti-Eomes, polyclonal, Cell Signaling Technology, Cat# 4540, dilution: 1:200 for ChIP
Anti-Blimp1 (clone C14A4), Cell Signaling Technology, Cat# 9115, dilution: 1:200 for ChIP
Rabbit IgG, polyclonal, Cell Signaling Technology, Cat# 2729, dilution: 1:200 for ChIP
Brilliant Violet 421™ anti-mouse CD8a Antibody (clone 53-6.7), BioLegend, Cat# 100738, dilution: 1:200
PE Donkey anti-rabbit IgG (minimal x-reactivity) Antibody, polyclonal, BioLegend, Cat# 406421, dilution: 1:1000
PerCP/Cyanine5.5 anti-mouse CD8a Antibody (clone 53-6.7), BioLegend, Cat# 100734, dilution: 1:200
Brilliant Violet 711™ anti-mouse CD45.1 Antibody (clone A20), BioLegend, Cat# 110739, dilution: 1:400
PE anti-mouse TIGIT (Vstm3) Antibody (clone 1G99), BioLegend, Cat# 142104, dilution: 1:400
PE/Cyanine7 anti-mouse CD279 (PD-1) Antibody (clone 29F.1A12), BioLegend, Cat# 135216, dilution: 1:400
PE anti-mouse TNF-α Antibody (clone MP6-XT22), BioLegend, Cat# 506306, dilution: 1:400
PE/Cyanine7 anti-mouse IFN-γ Antibody(clone XMG1.2), BioLegend, Cat# 505826, dilution: 1:400
Bim Rabbit mAb (Alexa Fluor® 488 Conjugate) (clone C34C5), Cell Signaling Technology, Cat# 94805, dilution: 1:400
Cleaved Caspase-3 (Asp175) Rabbit mAb (clone 5A1E), Cell Signaling Technology, Cat# 9664, dilution: 1:400
Brilliant Violet 421™ Donkey anti-rabbit IgG (minimal x-reactivity) Antibody, polyclonal, BioLegend, Cat# 406410, dilution: 1:1000
Ki-67 Antibody, anti-human/mouse, PE-Vio® 770, REAfinity™ (clone REA183), Miltenyi Biotec., Cat# 130-120-419, dilution: 1:400
Brilliant Violet 421™ anti-mouse CD4 Antibody (clone GK1.5), BioLegend, Cat# 100438, dilution: 1:200
APC/Cyanine7 anti-mouse CD4 Antibody (clone GK1.5), BioLegend, Cat# 100414, dilution: 1:200
Brilliant Violet 421™ anti-mouse/human CD44 Antibody (clone IM7), BioLegend, Cat# 103040, dilution: 1:400PE/Cyanine7 anti-mouse CD62L Antibody (clone MEL-14), BioLegend, Cat# 104418, dilution: 1:400
APC anti-mouse CD25 Antibody (clone 3C7), BioLegend, Cat# 101910, dilution: 1:400
PE anti-mouse CD127 (IL-7Rα) Antibody (clone A7R34), BioLegend, Cat# 135010, dilution: 1:400
Ultra-LEAF™ Purified anti-mouse CD16/32 Antibody (clone 93), BioLegend, Cat# 101330, dilution: 1:100
Ultra-LEAF™ Purified anti-mouse CD3 Antibody (clone 17A2), BioLegend, Cat# 100238, dilution: 2 ug/ml
Ultra-LEAF™ Purified anti-mouse CD28 Antibody (clone 37.51), BioLegend, Cat# 102116, dilution: 2 ug/ml
Ultra-LEAF™ Purified anti-human CD3 Antibody (clone OKT3), BioLegend, Cat# 317326, dilution: 2 ug/ml
Ultra-LEAF™ Purified anti-human CD2 Antibody (clone TS1/8), BioLegend, Cat# 309236, dilution: 2 ug/ml
Ultra-LEAF™ Purified anti-human CD28 Antibody (clone CD28.2), BioLegend, Cat# 302934, dilution: 2 ug/ml

Validation

Anti-GOT1 https://www.cellsignal.com/products/primary-antibodies/got1-e4a4o-rabbit-mab/34423
Alexa Fluor 488-conjugated donkey anti-rabbit secondary antibody https://www.biolegend.com/fr-lu/products/alexa-fluor-488-

donkey-anti-rabbit-igg-minimal-x-reactivity-9380
Alexa Fluor 647-conjugated anti-CD8a https://www.biolegend.com/en-gb/products/alexa-fluor-647-anti-human-cd8a-antibody-14127?GroupID=BLG15860
Anti-GRP94 https://www.cellsignal.com/products/primary-antibodies/grp94-d6x2q-xp-rabbit-mab/20292
Anti-NFAT1 https://www.cellsignal.com/products/primary-antibodies/nfat1-d43b1-xp-rabbit-mab/5861
Anti-TOX https://www.abcam.com/products/primary-antibodies/tox-antibody-ab155768.html
Anti-Eomes https://www.cellsignal.com/products/primary-antibodies/eomes-antibody/4540
Anti-Blimp1 https://www.cellsignal.com/products/primary-antibodies/blimp-1-prdi-bf1-c14a4-rabbit-mab/9115
Rabbit IgG https://www.cellsignal.com/products/primary-antibodies/normal-rabbit-igg/2729
Brilliant Violet 421™ anti-mouse CD8a Antibody https://www.biolegend.com/fr-fr/search-results/brilliant-violet-421-anti-mouse-cd8a-antibody-7138
PE Donkey anti-rabbit IgG (minimal x-reactivity) Antibody https://www.biolegend.com/fr-fr/products/pe-donkey-anti-rabbit-igg-minimal-x-reactivity-9751
PerCP/Cyanine5.5 anti-mouse CD8a Antibody https://www.biolegend.com/en-us/products/percp-cyanine5-5-anti-mouse-cd8a-antibody-4255
Brilliant Violet 711™ anti-mouse CD45.1 Antibody https://www.biolegend.com/en-us/products/brilliant-violet-711-anti-mouse-cd45-1-antibody-8925
PE anti-mouse TIGIT (Vstm3) Antibody https://www.biolegend.com/en-us/products/pe-anti-mouse-tigit-vstm3-antibody-7429
PE/Cyanine7 anti-mouse CD279 (PD-1) Antibody https://www.biolegend.com/en-us/products/pe-cyanine7-anti-mouse-cd279-pd-1-antibody-7005
PE anti-mouse TNF-α Antibody https://www.biolegend.com/en-us/products/pe-anti-mouse-tnf-alpha-antibody-978
PE/Cyanine7 anti-mouse IFN-γ Antibody https://www.biolegend.com/en-us/products/pe-cyanine7-anti-mouse-ifn-gamma-antibody-5865
Bim Rabbit mAb (Alexa Fluor® 488 Conjugate) https://www.cellsignal.com/products/antibody-conjugates/bim-c34c5-rabbit-mab-alexa-fluor-488-conjugate/94805
Cleaved Caspase-3 (Asp175) Rabbit mAb (clone 5A1E) https://www.cellsignal.com/products/primary-antibodies/cleaved-caspase-3-asp175-5a1e-rabbit-mab/9664
Brilliant Violet 421™ Donkey anti-rabbit IgG (minimal x-reactivity) Antibody https://www.biolegend.com/en-us/products/brilliant-violet-421-donkey-anti-rabbit-igg-minimal-x-reactivity-7262
Ki-67 Antibody, anti-human/mouse, PE-Vio® 770, REAfinity™ https://www.miltenyibiotec.com/DE-en/products/ki-67-antibody-anti-human-mouse-reafinity-rea183.html#conjugate=pe-vio-770:size=100-tests-in-200-ul
Brilliant Violet 421™ anti-mouse CD4 Antibody https://www.biolegend.com/en-us/products/brilliant-violet-421-anti-mouse-cd4-antibody-7142
APC/Cyanine7 anti-mouse CD4 Antibody https://www.biolegend.com/en-us/products/apc-cyanine7-anti-mouse-cd4-antibody-1964
Brilliant Violet 421™ anti-mouse/human CD44 Antibody https://www.biolegend.com/en-us/products/brilliant-violet-421-anti-mouse-human-cd44-antibody-7225
PE/Cyanine7 anti-mouse CD62L Antibody https://www.biolegend.com/en-us/products/pe-cyanine7-anti-mouse-cd62l-antibody-1922
APC anti-mouse CD25 Antibody https://www.biolegend.com/en-us/products/apc-anti-mouse-cd25-antibody-4512
PE anti-mouse CD127 (IL-7Rα) Antibody https://www.biolegend.com/en-us/products/pe-anti-mouse-cd127-il-7ralpha-antibody-6190
Ultra-LEAF™ Purified anti-mouse CD16/32 Antibody https://www.biolegend.com/en-us/products/ultra-leaf-purified-anti-mouse-cd16-32-antibody-8081
Ultra-LEAF™ Purified anti-mouse CD3 Antibody https://www.biolegend.com/en-us/products/ultra-leaf-purified-anti-mouse-cd3-antibody-8078
Ultra-LEAF™ Purified anti-mouse CD28 Antibody https://www.biolegend.com/en-us/products/ultra-leaf-purified-anti-mouse-cd28-antibody-7733
Ultra-LEAF™ Purified anti-human CD3 Antibody https://www.biolegend.com/en-us/products/ultra-leaf-purified-anti-human-cd3-antibody-7745
Ultra-LEAF™ Purified anti-human CD2 Antibody https://www.biolegend.com/en-us/search-results/ultra-leaf-purified-anti-human-cd2-antibody-19172?GroupID=BLG9913&gclid=CjwKCAjwoqGnBhAcEiwAwK-OkVkQoC2W8tSgy4348nJzJKM89IWKrggBopxNoAuUv8Ia8Eq2PbyAaxoCjUEQAvD_BwE
Ultra-LEAF™ Purified anti-human CD28 Antibody https://www.biolegend.com/en-us/products/ultra-leaf-purified-anti-human-cd28-antibody-7743

## Animals and other research organisms

Policy information about studies involving animals; ARRIVE guidelines recommended for reporting animal research, and Sex and Gender in Research

| | |
|---|---|
| Laboratory animals | Mice were maintained in the German cancer research center (DKFZ) specific pathogen-free facility. The Got1Flox/Flox mice, under the full name C57BL/6N-Got1tm1c(EUCOMM)Hmgu/H, were ordered from the MRC Harwell Institute, Oxfordshire, UK. Exon 2 of Got1 is flanked by two LoxP sites and is excised after crossing with a Cre-expression mouse strain. Cd4-Cre mice and P14 mice were from The Jackson Laboratory and have been backcrossed to C57BL/6N background for more than 10 generations. Mice were housed with a 12-h day–night cycle in a controlled environment at 20–24 °C and 45–65% humidity, and were fed a regular chow diet (cat#3437, Kliba Nafag) ad libitum. In rare cases, mice with fighting wounds were excluded from the experimental analysis. The sample collection and processing were not performed in a blinded manner. |
| Wild animals | We did not use wild animals. |
| Reporting on sex | We used both male and female mice in this study. We used sex-matched mice for each individual experiment. |
| Field-collected samples | No field-collected samples were used. |
| Ethics oversight | All studies were performed in accordance with DKFZ regulations with approval by the German regional council at the |

Ethics oversight | Regierungspräsidium Karlsruhe (G-232/16).

Note that full information on the approval of the study protocol must also be provided in the manuscript.

# Flow Cytometry

## Plots

Confirm that:

☒ The axis labels state the marker and fluorochrome used (e.g. CD4-FITC).

☒ The axis scales are clearly visible. Include numbers along axes only for bottom left plot of group (a 'group' is an analysis of identical markers).

☒ All plots are contour plots with outliers or pseudocolor plots.

☒ A numerical value for number of cells or percentage (with statistics) is provided.

## Methodology

Sample preparation

Spleens were smashed using a syringe through a 70 um cell strainer.
For surface antigen staining, Fc receptor blockers anti-CD16/CD32 were used to prevent nonspecific antibody binding. Cells were incubated in FACS buffer (PBS supplemented with 0.5% FCS) with the fluorescently conjugated antibodies for 30 minutes on ice. DAPI or a Live/DEAD Fixable Dead Cell Stain kit (Thermo Fisher) was used to exclude the dead cells. For intracellular cytokine staining, cells were fixed with the fixation buffer containing 4% paraformaldehyde (PFA; BioLegend) first, then permeabilized with eBioscience permeabilization buffer. For staining nuclear antigens, cells were fixed and permeabilized with the eBioscience Foxp3/transcription factor staining buffer set on ice for at least 30 minutes.

Instrument

Samples were washed and run on an LSR II or LSR Fortessa flow cytometer.

Software

We used FACS Diva Software (version 9, BD Biosciences) to collect FACS data and analyzed data in FlowJo software (10.1r1).

Cell population abundance

FACS sorting had a purity> 95%, which was confirmed by post-sort FACS analysis.

Gating strategy

Gates were first set based on FSC-A/SSC-A and doublets were excluded using FSC-H and FSC-A. Life/death dye was used to exclude dead cells. T cells were then gated for further analysis.

☒ Tick this box to confirm that a figure exemplifying the gating strategy is provided in the Supplementary Information.
