# Supplementary figures and images for: The malate shuttle detoxifies ammonia in exhausted T cells by producing 2-ketoglutarate
Source: Nat Immunol. 2023 Oct 9;24(11):1921–32. doi: 10.1038/s41590-023-01636-5 (PMC10602850; doi:10.1038/s41590-023-01636-5)

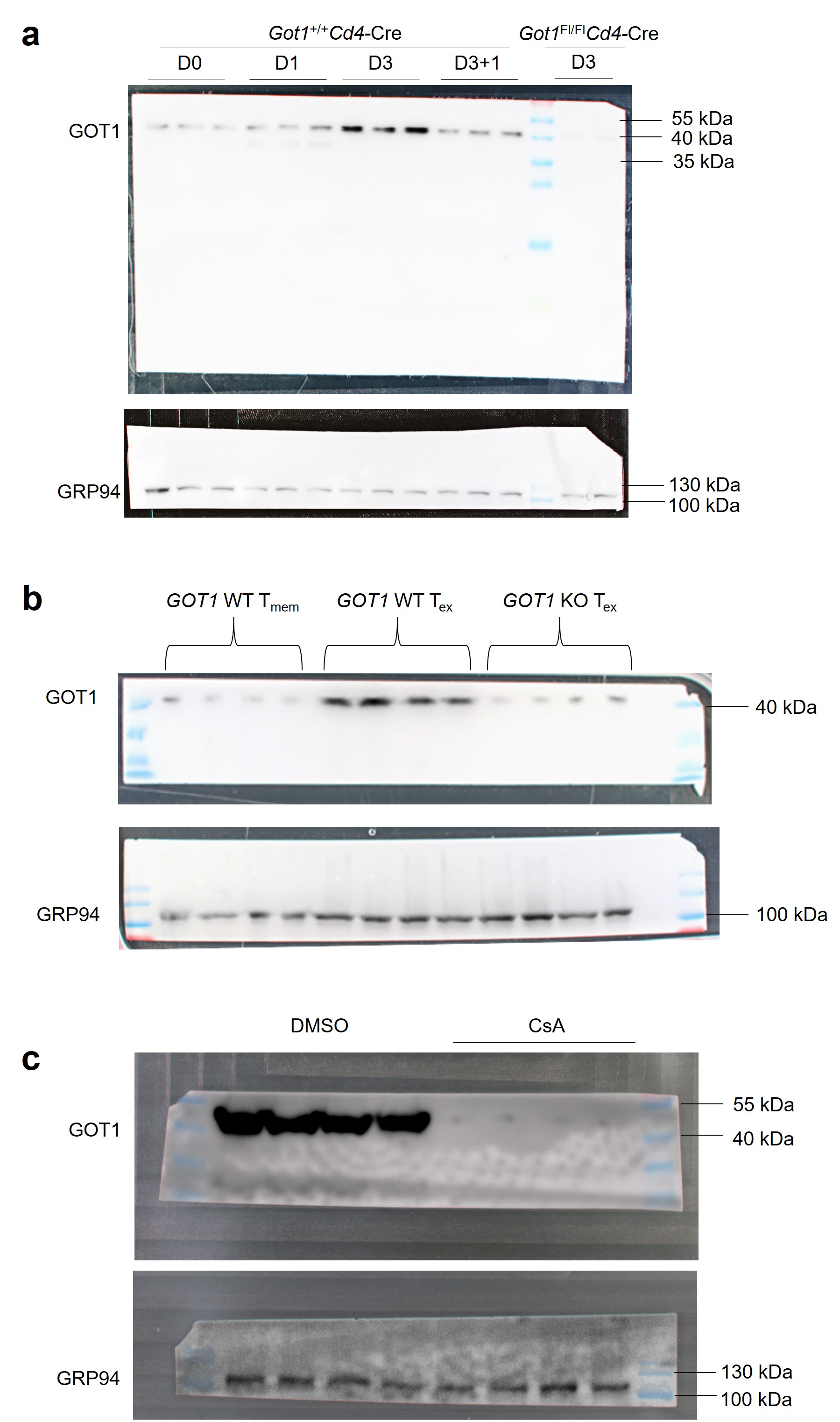

Supplement: Supplementary file 9 — Unprocessed western blots. [file 41590_2023_1636_MOESM9_ESM.jpg]
